# NEV supply chain coordination and sustainability considering sales effort and risk aversion under the CVaR criterion

**DOI:** 10.1371/journal.pone.0199005

**Published:** 2018-06-18

**Authors:** Shifeng Han, Xingzhong Xu

**Affiliations:** 1 School of Management and Economics, Beijing Institute of Technology, Beijing, China; 2 School of Mathematics and Statistics, Beijing Institute of Technology, Beijing, China; Central South University, CHINA

## Abstract

In a two-echelon new energy vehicle (NEV) supply chain consisting of a risk-neutral manufacturer and a risk-averse retailer, the coordination and sustainability problem is investigated. The risk-averse retailer, who makes sales effort and undertakes the incurred effort cost, decides the order quantity and sales effort level under the Conditional Value-at-Risk (CVaR) criterion. We derive the optimal centralized decisions of a vertically integrated supply chain where the retailer is owned by the manufacturer. Taking such a centralized case as the benchmark, we prove that the subsidy-sharing-based wholesale price (SS-WP) contract fails to coordinate the NEV supply chain under the decentralized case where the retailer makes decisions independently. Then we design a subsidy-sharing-based sales rebate/penalty (SS-SRP) contract and derive the contract parameters to achieve coordination. We evaluate the coordination efficiency of this contract and find that a well-designed SS-SRP contract can promote the NEV sales and lead to a Pareto-improving win-win situation for both the NEV manufacturer and retailer compared to the non-coordination case. A series of numerical experiments are carried out to compare the effects of significant parameters under the SS-WP and SS-SRP contract and provide additional observations and implications, including an indication of the necessary conditions to sustainably maintain the NEV supply chain.

## 1. Introduction

In the past decade, many countries have endeavored to develop new energy vehicles (NEVs) to cope with environmental deterioration and energy challenges. In China, the sustainable development of NEVs is extremely meaningful for improving air quality and adjusting the energy structure as well as promoting the reform and transformation of the automotive industry. But at present, most NEV managerial studies are qualitative research on policies or empirical research on consumers’ purchase intentions while quantitative research on NEV channel optimization and supply chain coordination is still rare. In the NEV commerce, a common practice is that the retailer orders from the manufacturer according to the demand prediction and makes sales effort trying to sell all the ordered NEVs. Under such a NEV business under the wholesale price contract, the retailer bears all the demand risk and the sales effort cost while the manufacturer bears all the high production cost. The NEV supply chain performance, including the order quantity and each player’s profit, cannot be optimized, which will be proved later in this paper. By a quantitative analysis on the NEV commerce, we aim to design a sustainable coordination mechanism for the NEV supply chain with an assumption that market demand is influenced by the retailer’s sales effort. The proposed coordination mechanism fully considers the peculiarities of the NEV industry, such as the extremely high production cost and the appearance of government subsidies. We evaluate the coordination efficiency and demonstrate that the proposed mechanism can sustainably promote the NEV sales and lead to a Pareto-improving win-win situation for both supply chain players.

With the marketization process of NEVs, consumers’ diversified demands are increasing. Meanwhile, government policies relating to NEVs are constantly adjusted, for example, the government subsidies have begun to decrease. In addition, as an emerging industry, NEVs’ market demand is very fluctuant. As the supply chain player directly facing consumers and the uncertain market demand, the retailer needs to bear great risk when making decisions on order quantity and sales effort level. Facing the increasing risk generated by the demand uncertainty, decision makers tend to be risk-averse rather than risk-neutral, which is supported by experimental results. Several studies, such as those by Eeckhoudt et al. (1995) [1], Agrawal and Seshadri (2000) [2], Chen et al. (2007) [3], and Shen et al. (2016) [4], have involved risk-averse decision makers using the newsvendor model by the traditional expected-utility method. All these studies imply that the optimal order (or production) quantity is reduced by risk aversion and decreases with respect to the risk aversion degree without consideration of the shortage penalty. The decision bias caused by risk aversion obviously makes challenges for the NEV supply chain coordination and sustainability. Our model considers the retailer as a risk-averse decision maker and attempts to explore the impact of risk aversion on the NEV supply chain coordination.

Besides the expected-utility method, there are three other major approaches being widely used in operations management to characterize risk aversion: mean-variance (MV) analysis (Markowitz, 1959) [5], Value-at-Risk (VaR) (Jorion, 1997) [6], and Conditional Value-at-Risk (CVaR) (Rockafellar and Uryasev, 2000, 2002) [7, 8]. MV analysis is an appropriate approach to model risk aversion for a class of decision makers with the concave quadratic utility function (see Buzacott et al., 2011 [9]; Chen and Federgruen, 2001 [10] for reviews). However, Ma et al. (2012) [11] explain that the MV approach is inadequate in the sense that it equally quantifies desirable upside outcomes and undesirable downside outcomes. In the VaR evaluation, the decision maker is allowed to specify a confidence level (say, *η* with *η* ∈ (0,1]) for attaining a certain level of wealth and we need to maximize the *η*-quantile of the profit function (Jorion, 1997) [6]. Since the VaR measure also has some limitations, such as nonsubadditivity and nonconvexity, Rockafellar and Uryasev (2000, 2002) [7, 8] define a new measure of risk, i.e., CVaR. The CVaR criterion, which measures the average profit falling below the *η*-quantile level (or VaR), has better computational characteristics than VaR (Artzner et al., 1999) [12]. Hence, recently more studies have investigated the risk-averse newsvendor problem in the CVaR framework (e.g., Ahmed et al., 2007 [13]; Gotoh and Takano, 2007 [14]; Choi and Ruszczynski, 2008 [15]; Chen et al., 2009 [16]). Due to the desirable mathematical characteristics, CVaR is adopted as the risk-averse retailer’s risky performance measurement in our model.

In this paper, we investigate the coordination and sustainability problem of a two-echelon NEV supply chain in which the market demand is influenced by the risk-averse retailer’s sales effort. It is supposed that the retailer makes a joint decision on order quantity and sales effort level with the objective of maximizing the risky performance measured by CVaR. In the centralized case where the retailer is owned by the manufacturer, we derive the decisions of the integrated supply chain. In the decentralized case where the retailer makes decisions independently, we prove that the subsidy-sharing-based wholesale price (SS-WP) contract fails to coordinate the NEV supply chain. To cope with such a problem, we propose an effective mechanism by combining the subsidy-sharing (SS) contract and the sales rebate/penalty (SRP) contract, and derive the contract parameters to achieve coordination. By evaluating the coordination efficiency, we analytically prove that the combined subsidy-sharing-based sales rebate/penalty (SS-SRP) contract with appropriate parameters can promote the NEV sales and enhance the profit of each supply chain player compared to the non-coordination case. Additionally, as the retailer is the only player who makes sales effort, we briefly discuss the free riding phenomenon in the supply chain. In particular, it is pointed out that the internal combustion engine vehicle (ICEV) supply chain is a special case of our model when the government subsidies disappear. By comparing the effects of significant parameters on the profit allocations under different contracts through a series of numerical experiments, we illustrate how to determine the sales target in the SS-SRP contract for the contract designer, how to set subsidies for the government, and the necessary conditions to sustainably maintain the NEV supply chain under the superior SS-SRP contract. The proposed model enriches research on the NEV supply chain coordination and sustainability by incorporating both the risk aversion effect and sales effort under the CVaR criterion. The designed coordination mechanism is proved to be effective, feasible and superior in promoting the NEV sales and enhancing each player’s profit. It provides guiding principles for the win-win cooperation between the NEV manufacturer and retailer as well as a suggestion for the government to set subsidies more appropriately, which can be beneficial to promote the sustainable development of the NEV commerce.

The rest of this paper is organized as follows. We review literature related to our work in Section 2. Section 3 provides a benchmark using the centralized model. In Section 4, we check the performance of the SS-WP contract and prove that it cannot coordinate the NEV supply chain. In Section 5, we propose a new SS-SRP coordination mechanism, evaluate the coordination efficiency, and analyze the free riding phenomenon. We carry out numerical experiments and obtain additional observations and implications in Section 6. In Section 7, we present some managerial insights according to the findings.

## 2. Literature review

The newsvendor model is regarded as a common method to characterize the risk aversion effect in operations management. Among the studies on the risk-averse newsvendor model, those using the CVaR criterion are more closely related to our work. For example, two early studies, Gotoh and Takano (2007) [14] and Chen et al. (2009) [16], investigate the risk-averse newsvendor problem under the CVaR criterion with the objective of minimizing CVaR about loss and maximizing CVaR about profit, respectively, and both studies demonstrate that risk aversion can reduce the newsvendor’s order quantity. Furthermore, recently CVaR has also been widely used in solving inventory problems in supply chains containing risk-averse players. For example, Cheng et al. (2009) [17] present a bilevel newsvendor model in a two-echelon system assuming that the retailer’s objective is to minimize the CVaR about loss and obtain an analytical solution when the product’s demand is uniformly distributed. Ma et al. (2012) [11] show that there exists a Nash-bargaining equilibrium about the wholesale price and order quantity between a risk-averse retailer and a risk-neutral supplier when the retailer tries to maximize the CVaR about profit. Due to the desirable mathematical characteristics, CVaR is also adopted by many researchers to model complex problems regarding risk-averse newsvendors. The related literature includes Xu (2010) [18], Xu and Li (2010) [19], Wu et al. (2014) [20], Luo et al. (2015) [21], Xue et al. (2015) [22], Xu et al. (2016) [23], and others. For example, CVaR is used to investigate the effects of parameter changes in the risk-averse newsvendor model by Xu (2010) [18], to characterize the optimal quantity and pricing decisions of a risk-averse newsvendor under both quantity and price competition by Wu et al. (2014) [20], and to formulate the opportunity loss of a risk-averse newsvendor by Xu et al. (2016) [23]. The appearance of a vast literature on the application of CVaR in the inventory problem indicates the accuracy and effectiveness of the CVaR approach. Even though these studies focus on different problems, all of them verify that a newsvendor’s order quantity is reduced by risk aversion and decreases with respect to the risk aversion degree. These studies on the newsvendor model, which incorporate risk aversion in different settings, make up the basis for designing the coordination mechanism of a supply chain containing a risk-averse player.

Coordinating a supply chain enables all players work together to maximize the total profit of the supply chain without reducing any player’s profit. There are numerous bodies of literature focusing on supply chain coordination (see Cachon, 2003 [24] for review). Most traditional studies design coordination mechanisms with an assumption that all players are risk-neutral and our research is more closely related to studies on supply chain coordination in a risk-averse setting. There are several studies that design applicable coordination mechanisms for supply chains containing risk-averse players. For example, Agrawal and Seshadri (2000) [2] investigate how to coordinate a supply chain which consists of a risk-neutral supplier and multiple risk-averse retailers. Gan et al. (2004) [25] propose a definition of supply chain coordination in a risk-averse setting according to the concept of Pareto-optimality. Gan et al. (2005) [26] develop a risk-sharing contract by adding a return policy to the initial contract to coordinate a supply chain consisting of a risk-neutral supplier and a risk-averse retailer. Yang et al. (2009) [27] address the supply chain with a risk-neutral supplier and a risk-averse retailer under the CVaR criterion and show how to coordinate the supply chain with the revenue-sharing, buy-back, two-part tariff and quantity flexibility contracts. These studies on supply chain coordination involve the risk aversion effect, but the designed mechanisms are not very practical in the automotive commerce. The reason is that the sales target, which is usually set in the automotive commerce, is not considered.

Among various supply chain coordination contracts, a class of contracts that set sales targets mostly conforms to the practice in the automotive commerce. For example, in the SRP contract, a sales target is set and when the selling season is over, the retailer obtains a rebate for the exceeding sales quantity or incurs a penalty for the insufficient sales quantity. Hu, Lim and Lu (2013) [28] provide a flexible ordering policy in a supply chain with random yield and demand uncertainty and propose a revenue-sharing policy with the SRP contract to achieve coordination. Giri, Bardhan and Maiti (2016) [29] combine buyback and SRP contracts to coordinate a three-layer supply chain containing one raw-material supplier, one manufacturer and one retailer. However, these studies about contracts that are applicable in the automotive commerce do not take the player’s risk attitude into account. It has been proved that it is not very appropriate to ignore the decision bias caused by risk aversion for deriving supply chain coordination conditions.

Our research is also related to the sales effort literature. Chu and Desai (1995) [30] show that the effective sales effort can improve the satisfaction degree of consumers and further expand the market demand. Consequently, it is necessary to investigate the coordination problem of a supply chain where the demand is relevant to sales effort level. There has been research incorporating sales effort in different settings or under different contracts to evaluate supply chain performance. Taylor (2002) [31] designs a combination of buyback contract and discount contract with a sales target set to coordinate a supply chain in which the market demand is influenced by sales effort. Based on Taylor’s work, Krishnan, Kapuscinski and Butz (2004) [32] suppose that a retailer can make a decision on sales effort level after observing the market demand, and they prove that the SRP contract can coordinate this supply chain. He et al. (2009) [33] provide a combination of contracts based on quantity discount and penalty to coordinate the supply chain under the setting that demand is jointly influenced by sales effort level and price. Kumar and Ruan (2006) [34] analyze a dual-channel supply chain considering the effect of sales effort on demand and discuss the difference between the traditional retail channel and the online channel. Pu, Gong and Han (2017) [35] explore the free riding problem in a dual-channel supply chain and show that the coordination situation can be achieved by the way players share the sales effort cost. These studies consider how the sales effort level affects supply chain coordination, but they all assume a risk-neutral environment rather than a risk-averse one, and the corresponding results are not appropriate for the NEV supply chain.

The application background of our work is the NEV commerce. As an emerging industry, NEVs are paid much attention by many governments. Research on NEVs not only focuses on battery technology but also emphasizes marketing and consumer behavior. Bapna, Thakur and Nair (2002) [36] suggest that governments should improve charging facilities to decrease usage costs of NEVs for consumers; Wang, Pan and Zheng (2017) [37] use a multiple linear regression method to identify four key factors that promote NEV sales; Zhang and Bai (2017) [38] propose a policy-dependency mapping method to analyze 175 government policies regarding NEVs at various levels with multiple purposes. These qualitative studies show that there are several policies that can promote the advancement of NEV commerce, such as strengthening research and development, establishing specific subsidies and tax policies, improving charging facilities, and so on. There are also some quantitative studies addressing the NEV supply chain or NEV commerce. Luo et al. (2014) [39] quantitatively investigate the NEV supply chain under a government’s price-discount incentive scheme that involves a price discount rate and a subsidy ceiling. They derive the most effective discount rate and subsidy ceiling that maximize the NEV sales as well as that most effectively improve the manufacturer’s incentive for NEV production. Liu, Huang and Yang (2017) [40] build an evolutionary game model between NEV manufacturers and governments and find that the evolutionary game exhibits to be stable when governments implement a dynamic taxation strategy or a dynamic subsidy strategy. The simulation on China’s NEV industry indicates that a policy of dynamic taxations and static subsidies is more effective for the NEV industry development. Shao, Yang and Zhang (2017) [41] address the NEV market under two different structures (monopoly and duopoly) and formulate a utility model composed of a population of consumers who make utility maximizing choices and manufacturers who set an optimal pricing. They show that the government prefers to implement a subsidy incentive scheme rather than a price discount incentive scheme and under the subsidy incentive scheme, the NEV market has a smaller environmental impact in the monopoly setting than in the duopoly setting. In particular, two other papers involve a behavioral element, loss aversion, to explore the NEV optimal production strategy under risk. Zhang (2014) [42] considers both consumer trade-offs and government subsidies together with the loss aversion of decision makers to evaluate relevant influences on the NEV optimal production strategy and indicates that subsidies can help to increase the NEV production quantity and offset the loss aversion effect. Gu, Liu and Qing (2017) [43] investigate a loss-averse NEV manufacturer’s optimal production decision considering battery recycling and prove that battery recycling can offset the negative effect of loss aversion on the optimal production quantity and expected utility. However, studies on the NEV supply chain considering another behavioral element, risk aversion, are quite scarce. Apart from all the mentioned studies, we introduce risk aversion under the CVaR criterion, propose a quantitative model, and focus on the NEV supply chain coordination and sustainability with full consideration of its peculiarities, the high production cost and the appearance of government subsidies. By designing a coordination mechanism for the NEV supply chain, we hope to provide some insights into implications for the win-win cooperation between the NEV manufacturer and retailer, which may help to sustainably promote the NEV marketization.

## 3. Benchmark: The centralized model

To provide an efficiency benchmark, we start our analysis with the centralized case. Suppose that a NEV supply chain consists of one retailer and one manufacturer (hereafter we use he/his to stand for the retailer/retailer’s and she/her for the manufacturer/manufacturer’s). The supply chain faces a stochastic demand and sells NEVs to consumers. To promote the NEV sales, the retailer makes a certain level of sales effort *e* before the selling season. The retailer hence incurs a corresponding sales effort cost *V*(*e*) = *θe*^2^/2, where *θ* represents the sales effort cost coefficient (Taylor, 2002 [31]), *θ* > 0. The random market demand *x* is influenced by the retailer’s sales effort level *e* and thus has a probability density function (*p*.*d*.*f*.) of *f*(*x*|*e*) and a cumulative distribution function (*c*.*d*.*f*.) of *F*(*x*|*e*). We assume the market demand is in additive form with effort-dependent demand, i.e., *x* = *z*(e)+*ξ*, where *z*(*e*) is an increasing nonconvex function standing for the part of demand with respect to *e* and *ξ* is the stochastic part with a *p*.*d*.*f* of *ϕ*(*ξ*) and a *c*.*d*.*f*. of Φ(*ξ*). Suppose that Φ(*ξ*) is invertible and *ϕ*(*ξ*) has a continuous first-order derivative *ϕ*′(*ξ*). Then, we have *f*(*x*|*e*) = *ϕ*(*x* − *z*(*e*)) and *F*(*x*|*e*) = Φ(*x* − *z*(*e*)).

Suppose NEVs are produced at a unit cost of *c* and sold to consumers at an exogenous price *p*. At the end of the selling season, the unsatisfied demand will be lost and the leftover inventory will be disposed at a unit salvage value *s*. Assume that *c* > *p* > *s*. Such an assumption conforms to the practice in the NEV industry because the NEV production cost is extremely high (especially the battery cost), even higher than the retail price. Since the NEV manufacturer and retailer cannot afford the high production cost independently, NEVs are subsidized by both national and local governments. We denote the per unit amount of government subsidies as *Y*, and assume that *p* + *Y* > *c* which ensures that the NEV supply chain can obtain positive profit from the perspective of system with the government’s support.

Suppose a centralized case where the NEV retailer is owned by the NEV manufacturer and thus the NEV supply chain is vertically integrated. The optimal solutions of the centralized supply chain, which do not depend on the contracts between players, can be considered as the benchmark. In such a centralized case, the NEV manufacturer acts as the central planner and decides the optimal delivery quantity and sales effort level with the objective of maximizing the total expected profit of the integrated supply chain. The manufacturer faces a newsvendor problem in which the market demand is *x*, and intends to decide the delivery quantity *q*_*s*_ and the sales effort level *e*_*s*_ to obtain maximum profit.

The profit and expected profit of the integrated supply chain can be expressed as *π*_*s*_(*q*_*s*_, *e*_*s*_) and *Eπ*_*s*_(*q*_*s*_, *e*_*s*_), respectively.

$$\begin{array}{ll} \pi_{s}\left(q_{s},e_{s}\right) & =\{\begin{array}{ll} \left(p+Y\right)x-cq_{s}+s\left(q_{s}-x\right)-\theta e_{s}{}^{2}/2 & x\leq q_{s}\\ \left(p+Y\right)q_{s}-cq_{s}-\theta e_{s}{}^{2}/2 & x>q_{s} \end{array}\\ & =\{\begin{array}{ll} \left(p+Y-s\right)x-\left(c-s\right)q_{s}-\theta e_{s}{}^{2}/2 & x\leq q_{s}\\ \left(p+Y-c\right)q_{s}-\theta e_{s}{}^{2}/2 & x>q_{s} \end{array} \end{array}$$

$$\begin{array}{ll} E\pi_{s}\left(q_{s},e_{s}\right) & =\int_{0}^{q_{s}}\left[\left(p+Y-s\right)x-\left(c-s\right)q_{s}-\theta e_{s}{}^{2}/2\right]dF(x|e_{s})\\ & +\int_{q_{s}}^{+\infty }\left[\left(p+Y-c\right)q_{s}-\theta e_{s}{}^{2}/2\right]dF(x|e_{s})\\ & =\left(p+Y-c\right)q_{s}-\left(p+Y-s\right)\int_{0}^{q_{s}}F(x|e_{s})dx-\theta e_{s}{}^{2}/2 \end{array}$$ (3.1)

Basing on the above results, we provide the following theorem about the manufacturer’s centralized decisions on the delivery quantity and the sales effort level.

**Theorem 3.1.** *The optimal centralized joint decision of the NEV supply chain is $\left(q_{s}^{*},e_{s}^{*}\right)$, where*

$$q_{s}^{*}=\Phi^{-1}\left(\frac{p+Y-c}{p+Y-s}\right)+z\left(e_{s}^{*}\right)$$ (3.2)

$$\left(p+Y-c\right)z\prime \left(e_{s}^{*}\right)-\theta e_{s}^{*}=0$$ (3.3)

**Proof.** Refer to Appendix A1.

Theorem 3.1 provides a benchmark for the subsequent analysis. When all related parameters are given, the optimal delivery quantity and sales effort level of the integrated supply chain can be calculated according to Eqs (3.2) and (3.3). The joint decision $\left(q_{s}^{*},e_{s}^{*}\right)$ can maximize the total expected profit of the supply chain, and the corresponding maximum expected profit is calculated and presented as follows.

$$E\pi_{s}(q_{s}^{*},e_{s}^{*})=\left(p+Y-c\right)z\left(e_{s}^{*}\right)+\left(p+Y-s\right)\int_{0}^{\Phi^{-1}\left(\frac{p+Y-c}{p+Y-s}\right)}\xi d\Phi \left(\xi \right)-\theta e_{s}^{*2}/2$$ (3.4)

From Eqs (3.2) and (3.3), we can easily find that the optimal delivery quantity and sales effort level of the supply chain both increase in price *p* and decreases in production cost *c*. These conclusions are straightforward and intuitive, so we omit detailed discussions about them. However, an increase of the salvage value *s* can increase the delivery quantity while having no effect on the sales effort level. If *s* increases, the losses brought by the leftover inventory decrease, so the delivery quantity will be increased. On the other hand, for arbitrary *s* < *p*, a NEV sold to consumer is more beneficial than a leftover one for the supply chain so that the optimal sales effort level should be determined to expand the market demand and increase sales as much as possible. Consequently, the optimal sales effort decision just concerns how to balance the profit generated by the sales effort and the incurred cost while has nothing to do with *s*.

Because the appearance of government subsidies is a peculiarity in the NEV industry, we are interested in how subsidies influence the NEV supply chain’s optimal solutions. The following proposition reveals the relationship between the centralized decisions and government subsidies.

**Proposition 3.1.** *The optimal delivery quantity and sales effort level both increase with respect to government subsidies*.

**Proof.** Refer to Appendix A2.

Proposition 3.1 indicates that when government subsidies increase, the integrated NEV supply chain tends to make more sales efforts and deliver more NEVs. Therefore, we conclude that government subsidies can induce the supply chain to increase the sales effort level and promote NEV sales in the centralized case. Such a conclusion is consistent with previous research on the effect of NEV government subsidies. However, as a profit source outside the NEV supply chain, government subsidies act as the dependence for the supply chain, accompanied by a positive effect. If the purpose of making more sales efforts and promoting sales is to get more subsidies from the government, it may bring about a negative effect on the sustainable development of the NEV supply chain.

## 4. Non-coordination contract example: SS-WP contract

Suppose that the NEV manufacturer is risk-neutral and the NEV retailer is risk-averse. In the decentralized case, the retailer makes decisions on the order quantity and sales effort level with the objective of maximizing his risky performance measured by CVaR without considering the profit of the manufacturer or the entire supply chain. In order to maximize the supply chain’s profit, a coordination mechanism should ensure that the retailer makes individual decisions aligning with the optimal centralized solutions of the integrated supply chain. Cachon (2003) [24] indicates that most of general coordination contracts, including the WP contract, buyback contract, revenue sharing contract and others, all fail to coordinate a supply chain with effort-dependent stochastic demand. In this section, we discuss the performance of the SS-WP contract, which provides an example of non-coordination contracts and serves as the comparison object for the efficiency evaluation of the proposed coordination contract.

Since the single WP contract can be considered as a special case of the SS-WP contract in which the manufacturer occupies all the government subsidies rather than sharing them with the retailer, we involve the SS policy to provide a more generalized model. Denote the SS-WP contract as (*w*_*w*_, *β*_*w*_) where *w*_*w*_ is the wholesale price, and *β*_*w*_ is the SS proportion for the NEV manufacturer and the remaining (1 − *β*_*w*_) proportion belongs to the retailer.

In the decentralized case, *q*_*w*_, *e*_*w*_ and *π*_*r*_(*q*_*w*_, *e*_*w*_) represent the retailer’s order quantity, sales effort level and profit under the SS-WP contract (*w*_*w*_, *β*_*w*_), respectively. *π*_*r*_(*q*_*w*_, *e*_*w*_) can be expressed as follows.

$$\begin{array}{ll} \pi_{r}\left(q_{w},e_{w}\right) & =\left[p+\left(1-\beta_{w}\right)Y\right]\text{min}\left(q_{w},x\right)-w_{w}q_{w}+s{\left(q_{w}-x\right)}^{+}-\theta e_{w}^{2}/2\\ & =\left[p+\left(1-\beta_{w}\right)Y-w_{w}\right]\text{min}\left(q_{w},x\right)-\left(w_{w}-s\right){\left(q_{w}-x\right)}^{+}-\theta e_{w}^{2}/2\\ & =\left[p+\left(1-\beta_{w}\right)Y-w_{w}\right]\left[q_{w}-{\left(q_{w}-x\right)}^{+}\right]-\left(w_{w}-s\right){\left(q_{w}-x\right)}^{+}-\theta e_{w}^{2}/2\\ & =\left[p+\left(1-\beta_{w}\right)Y-w_{w}\right]q_{w}-\left[p+\left(1-\beta_{w}\right)Y-s\right]{\left(q_{w}-x\right)}^{+}-\theta e_{w}^{2}/2 \end{array}$$ (4.1)

The risk-averse retailer intends to determine the optimal order quantity $q_{w}^{*}$ and sales effort level $e_{w}^{*}$ with the objective of maximizing CVaR. The problem can be formulated as follows.

$$\underset{q_{w}\geq 0,e_{w}\geq 0}{\text{max}}\;\;\;\;\;\;CVaR\left(\pi_{r}\left(q_{w},e_{w}\right)\right)$$ (4.2)

For calculating convenience, we adopt the following definition of *CVaR* (see Rockafellar and Uryasev, 2000 [7]; Chen et al., 2009 [16]).

$$\begin{array}{ll} CVaR\left(\pi_{r}\left(q_{w},e_{w}\right)\right) & =\underset{v_{w}\in R}{\text{max}}\;\left\{v_{w}+\frac{1}{\eta }E\left[\text{min}\left(\pi_{r}\left(q_{w},e_{w}\right)-v_{w},0\right)\right]\right\}\\ & =\underset{v_{w}\in R}{\text{max}}\;\left\{v_{w}-\frac{1}{\eta }E{\left[v_{w}-\pi_{r}\left(q_{w},e_{w}\right)\right]}^{+}\right\} \end{array}$$ (4.3)

where *E* is the expectation operator, (·)^+^ = max{·, 0}, *v*_*w*_ is a threshold denoted in the real number set *R*, and *η* is the confidence level of the retailer. The lower *η* is, the more risk-averse the retailer is. Based on these definitions and denotations, in order to solve the problem (4.2), we provide the following theorem to show the CVaR expression of the retailer under the SS-WP contract.

**Theorem 4.1.** *The risk-averse retailer’s CVaR under the SS-WP contract can be expressed as follows*.

$$CVaR\left(\pi_{r}\left(q_{w},e_{w}\right)\right)=\{\begin{array}{ll} \left[p+\left(1-\beta_{w}\right)Y-w_{w}\right]q_{w}-\frac{p+\left(1-\beta_{w}\right)Y-s}{\eta }\int_{0}^{q_{w}}F\left(x|e_{w}\right)dx-\theta e_{w}^{2}/2 & q_{w}\leq F^{-1}\left(\eta |e_{w}\right)\\ \left[p+\left(1-\beta_{w}\right)Y-s\right]F^{-1}\left(\eta |e_{w}\right)-\left(w_{w}-s\right)q_{w}-\frac{p+\left(1-\beta_{w}\right)Y-s}{\eta }\int_{0}^{F^{-1}\left(\eta |e_{w}\right)}F\left(x|e_{w}\right)dx-\theta e_{w}^{2}/2 & q_{w}>F^{-1}\left(\eta |e_{w}\right) \end{array}$$ (4.4)

**Proof.** Refer to Appendix A3.

Theorem 4.1 explicitly provides the expression of the risk-averse NEV retailer’s CVaR under the SS-WP contract. With all variables given, if the retailer’s ordering quantity is *q*_*w*_ and the sales effort level is *e*_*w*_, the CVaR the retailer achieves, denoted as *CVaR*(*π*_*r*_(*q*_*w*_, *e*_*w*_)), can be calculated using Eq (4.4). Considering the problem (4.2), the risk-averse retailer intends to determine the optimal order quantity and sales effort level with the objective of maximizing *CVaR*(*π*_*r*_(*q*_*w*_, *e*_*w*_)). According to the CVaR expression provided by Theorem 4.1, we can obtain the retailer’s optimal decisions under the SS-WP contract, shown in the following proposition.

**Proposition 4.1.** *Under the SS-WP contract, the optimal joint decision of the risk-averse retailer is* $\left(q_{w}^{*},e_{w}^{*}\right)$, *where*

$$q_{w}^{*}=\Phi^{-1}\left(\frac{\eta \left[p+\left(1-\beta_{w}\right)Y-w_{w}\right]}{p+\left(1-\beta_{w}\right)Y-s}\right)+z\left(e_{w}^{*}\right)$$ (4.5)

$$\left[p+\left(1-\beta_{w}\right)Y-w_{w}\right]z\prime \left(e_{w}^{*}\right)-\theta e_{w}^{*}=0$$ (4.6)

**Proof.** Refer to Appendix A4.

Proposition 4.1 provides the risk-averse NEV retailer’s joint decision on the order quantity and sales effort level $\left(q_{w}^{*},e_{w}^{*}\right)$ under the SS-WP contract. With an adequate consideration of the risk arising from the demand uncertainty, $\left(q_{w}^{*},e_{w}^{*}\right)$ is obtained with the objective of maximizing CVaR measurement. When the retailer’s confidence level and other parameters are given, the unique optimal order quantity and sales effort level can be calculated by Eqs (4.5) and (4.6).

In order to maximize the supply chain’s profit, the two parameters in the SS-WP contract (*w*_*w*_, *β*_*w*_) should be set trying to align the risk-averse retailer’s individual decisions with the optimal centralized decisions of the integrated supply chain. Comparing the results in Theorem 3.1 and Proposition 4.1, we provide the following proposition to show the performance of the SS-WP contract in coordinating the NEV supply chain.

**Proposition 4.2.** *The SS-WP contract cannot coordinate the NEV supply chain containing a risk-averse retailer and facing an effort-dependent demand*.

**Proof.** Refer to Appendix A5.

For the NEV supply chain which contains a risk-averse retailer and has an effort-dependent demand, under the SS-WP contract, the conditions which can align the retailer’s individual decisions with the centralized decisions of the integrated supply chain will lead to zero profit for the manufacturer. Since increasing the sales effort level can enhance the market demand, the retailer can take the initiative in the supply chain by enlarging the sales effort level and order quantity. When the sales effort level and order quantity are both equal to the supply chain’s centralized decisions, the retailer occupies all the profit of the supply chain by enough sales effort. The manufacturer cannot obtain any profit in such a situation and therefore does not participate in the business. Thus, the supply chain cannot be coordinated by the SS-WP contract.

Although the SS-WP contract cannot optimize the supply chain’s total profit, the NEV supply chain may be maintained under the SS-WP contract when *w*_*w*_ and *β*_*w*_ are appropriately modified to satisfy *w*_*w*_ + *β*_*w*_*Y* − *c* > 0 and *p* + (1 − *β*_*w*_)*Y* − *w*_*w*_ > 0. Under such a situation, both the manufacturer and the retailer can obtain positive profits from the business and therefore may accept the contract. For example, if the wholesale price *w*_*w*_ is fixed at *c* − (1 − *η*) (*p* + *Y* − *s*), *β*_*w*_ should be enhanced to a level higher than (1 − *η*_*r*_) (*p* + *Y* − *s*)/*Y*. At this time, although the supply chain’s total profit is not maximized, the manufacturer can obtain positive profit. When *β*_*w*_ is high enough, the manufacturer will have the aspiration to participate in such a business.

In order to compare the profit allocation under different contracts later in the numerical experiments, we next calculate the expected profits of the retailer and the manufacturer under the SS-WP contract. According to Eq (4.1), the retailer’s expected profit can be expressed as follows.

$$\begin{array}{ll} E\pi_{r}(q_{w},e_{w}) & =\int_{0}^{q_{w}}\left[\left[p+\left(1-\beta_{w}\right)Y-s\right]x-\left(w_{w}-s\right)q_{w}-\theta e_{w}^{2}/2\right]dF(x|e_{w})\\ & +\int_{q_{w}}^{+\infty }\left[\left[p+\left(1-\beta_{w}\right)Y-w_{w}\right]q_{w}-\theta e_{w}^{2}/2\right]dF(x|e_{w})\\ & =\left[\left[p+\left(1-\beta_{w}\right)Y-w_{w}\right]q_{w}-\theta e_{w}^{2}/2\right]F(q_{w}|e_{w})-\left[p+\left(1-\beta_{w}\right)Y-s\right]\int_{0}^{q_{w}}F(x|e_{w})dx\\ & +\left[\left[p+\left(1-\beta_{w}\right)Y-w_{w}\right]q_{w}-\theta e_{w}^{2}/2\right]\left[1-F(q_{w}|e_{w})\right]\\ & =\left[p+\left(1-\beta_{w}\right)Y-w_{w}\right]q_{w}-\left[p+\left(1-\beta_{w}\right)Y-s\right]\int_{0}^{q_{w}}F(x|e_{w})dx-\theta e_{w}^{2}/2 \end{array}$$

With the risk-averse retailer’s CVaR-maximizing decision $\left(q_{w}^{*},e_{w}^{*}\right)$ shown in Proposition 4.2, the retailer’s expected profit can be expressed as follows.

$$\begin{array}{ll} E\pi_{r}(q_{w}^{*},e_{w}^{*}) & =\left[p+\left(1-\beta_{w}\right)Y-w_{w}\right]q_{w}^{*}-\left[p+\left(1-\beta_{w}\right)Y-s\right]\int_{0}^{q_{w}^{*}}F(x|e_{w}^{*})dx-\theta e_{w}^{*2}/2\\ & =\left[p+\left(1-\beta_{w}\right)Y-w_{w}\right]\left[\Phi^{-1}\left(\frac{\eta \left[p+\left(1-\beta_{w}\right)Y-w_{w}\right]}{p+\left(1-\beta_{w}\right)Y-s}\right)+z\left(e_{w}^{*}\right)\right]-\left[p+\left(1-\beta_{w}\right)Y-s\right]\int_{0}^{\Phi^{-1}\left(\frac{\eta \left[p+\left(1-\beta_{w}\right)Y-w_{w}\right]}{p+\left(1-\beta_{w}\right)Y-s}\right)+z\left(e_{w}^{*}\right)}\Phi \left(x-z\left(e_{w}^{*}\right)\right)dx-\theta e_{w}^{*2}/2\\ & =\left[p+\left(1-\beta_{w}\right)Y-w_{w}\right]\left[\Phi^{-1}\left(\frac{\eta \left[p+\left(1-\beta_{w}\right)Y-w_{w}\right]}{p+\left(1-\beta_{w}\right)Y-s}\right)+z\left(e_{w}^{*}\right)\right]-\left[p+\left(1-\beta_{w}\right)Y-s\right]\int_{0}^{\Phi^{-1}\left(\frac{\eta \left[p+\left(1-\beta_{w}\right)Y-w_{w}\right]}{p+\left(1-\beta_{w}\right)Y-s}\right)}\Phi \left(\xi \right)d\xi -\theta e_{w}^{*2}/2 \end{array}$$ (4.7)

Similarly, the manufacturer’s expected profit under the SS-WP contract, *Eπ*_*m*_(*q*_*w*_, *e*_*w*_), is formulated as follows.

$$\begin{array}{ll} E\pi_{m}(q_{w},e_{w}) & =\int_{0}^{q_{w}}\left[\left(w_{w}-c\right)q_{w}+\beta_{w}Yx\right]dF(x|e_{w})\\ & +\int_{q_{w}}^{+\infty }\left[\left(w_{w}+\beta_{w}Y-c\right)q_{w}\right]dF(x|e_{w})\\ & =\left[\left(w_{w}-c\right)q_{w}+\beta_{w}Yq_{w}\right]F(q_{w}|e_{w})-\beta_{w}Y\int_{0}^{q_{w}}F(x|e_{w})dx\\ & +\left[\left(w_{w}+\beta_{w}Y-c\right)q_{w}\right]\left[1-F(q_{w}|e_{w})\right]\\ & =\left(w_{w}+\beta_{w}Y-c\right)q_{w}-\beta_{w}Y\int_{0}^{q_{w}}F(x|e_{w})dx \end{array}$$

With the retailer’s decision $\left(q_{w}^{*},e_{w}^{*}\right)$, the manufacturer’s expected profit can be obtained.

$$\begin{array}{ll} E\pi_{m}(q_{w}^{*},e_{w}^{*}) & =\left(w_{w}+\beta_{w}Y-c\right)q_{w}^{*}-\beta_{w}Y\int_{0}^{q_{w}^{*}}F(x|e_{w}^{*})dx\\ & =\left(w_{w}+\beta_{w}Y-c\right)\left[\Phi^{-1}\left(\frac{\eta \left[p+\left(1-\beta_{w}\right)Y-w_{w}\right]}{p+\left(1-\beta_{w}\right)Y-s}\right)+z\left(e_{w}^{*}\right)\right]-\beta_{w}Y\int_{0}^{\Phi^{-1}\left(\frac{\eta \left[p+\left(1-\beta_{w}\right)Y-w_{w}\right]}{p+\left(1-\beta_{w}\right)Y-s}\right)+z\left(e_{w}^{*}\right)}\Phi \left(x-z\left(e_{w}^{*}\right)\right)dx\\ & =\left(w_{w}+\beta_{w}Y-c\right)\left[\Phi^{-1}\left(\frac{\eta \left[p+\left(1-\beta_{w}\right)Y-w_{w}\right]}{p+\left(1-\beta_{w}\right)Y-s}\right)+z\left(e_{w}^{*}\right)\right]-\beta_{w}Y\int_{0}^{\Phi^{-1}\left(\frac{\eta \left[p+\left(1-\beta_{w}\right)Y-w_{w}\right]}{p+\left(1-\beta_{w}\right)Y-s}\right)}\Phi \left(\xi \right)d\xi \end{array}$$ (4.8)

## 5. SS-SRP coordination contract

As the SS-WP contract cannot coordinate the NEV supply chain, we propose a combined SS-SRP contract and determine appropriate parameters to achieve coordination. In this section, we first provide the risk-averse retailer’s individual decisions on the order quantity and sales effort level under the SS-SRP contract. By comparing the decentralized decisions with the centralized decisions of the integrated supply chain, we derive the parameters of the coordination contract. Then we evaluate the coordination efficiency and analyze the effectiveness of the proposed contract.

### 5.1 The retailer’s decisions under the SS-SRP contract

**Fig 1:**
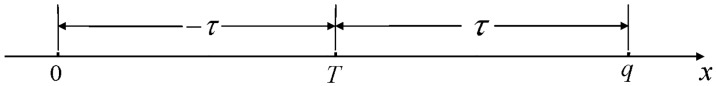
The SS-SRP contract faced by retailer.

Suppose an SS-SRP contract (*w*, *τ*, *β*, *T*) is adopted in the NEV supply chain. Here *w* is the wholesale price, *τ* is the unit rebate/penalty, *β* is the SS proportion for the NEV manufacturer and the remaining (1 − *β*) proportion belonging to the retailer, and *T* is the sales target. When the selling season is over, the predetermined contract is executed: the retailer obtains a unit rebate *τ* for the exceeding sales quantity over *T* from the manufacturer or pays the same amount of unit penalty *τ* for the insufficient sales quantity under *T* to the manufacturer. In addition, the government subsidies are divided by the manufacturer and retailer with the proportion *β*:(1 − *β*). In the decentralized case, denote *q* (*q* > *T*), *e* and *π*_*r*_(*q*, *e*) to respectively represent the retailer’s order quantity, sales effort level and profit under the SS-SRP contract. As the decision maker in the decentralized case, the retailer faces an SS-SRP contract shown in Fig 1.

According to the above assumptions, the retailer’s profit *π*_*r*_(*q*, *e*) can be expressed as follows.

$$\begin{array}{ll} \pi_{r}\left(q,e\right) & =\left[p+\left(1-\beta \right)Y\right]\text{min}\left(q,x\right)-wq+s{\left(q-x\right)}^{+}+\tau \left[\text{min}\left(q,x\right)-T\right]-\theta e^{2}/2\\ & =\left[p+\left(1-\beta \right)Y-w+\tau \right]\text{min}\left(q,x\right)-\left(w-s\right){\left(q-x\right)}^{+}-\left(\tau T+\theta e^{2}/2\right)\\ & =\left[p+\left(1-\beta \right)Y-w+\tau \right]\left[q-{\left(q-x\right)}^{+}\right]-\left(w-s\right){\left(q-x\right)}^{+}-\left(\tau T+\theta e^{2}/2\right)\\ & =\left[p+\left(1-\beta \right)Y-w+\tau \right]q-\left[p+\left(1-\beta \right)Y-s+\tau \right]{\left(q-x\right)}^{+}-\left(\tau T+\theta e^{2}/2\right) \end{array}$$ (5.1)

The risk-averse retailer intends to determine the optimal order quantity q* and sales effort level e* with the objective of maximizing CVaR. The problem can be formulated as follows.

$$\underset{q\geq 0,e\geq 0}{\text{max}}\;\;\;\;\;\;CVaR\left(\pi_{r}\left(q,e\right)\right)$$ (5.2)

Similar to Eq (4.3), *CVaR*(*π*_*r*_(*q*, *e*)) can be formulated as follows.

$$\begin{array}{ll} CVaR\left(\pi_{r}\left(q,e\right)\right) & =\underset{v\in R}{\text{max}}\;\left\{v+\frac{1}{\eta }E\left[\text{min}\left(\pi_{r}\left(q,e\right)-v,0\right)\right]\right\}\\ & =\underset{v\in R}{\text{max}}\;\left\{v-\frac{1}{\eta }E{\left[v-\pi_{r}\left(q,e\right)\right]}^{+}\right\} \end{array}$$ (5.3)

where *E* is the expectation operator, (·)^+^ = max{·, 0}, *v* is a threshold denoted in the real number set *R*, and *η* is the confidence level of the retailer. Based on these definitions and denotations, in order to solve problem (5.2), we provide the following theorem to show the CVaR expression of the retailer under the SS-SRP contract.

**Theorem 5.1.** *The risk-averse retailer’s CVaR under the SS-SRP contract can be expressed as follows*.

$$CVaR\left(\pi_{r}\left(q,e\right)\right)=\{\begin{array}{ll} \left[p+\left(1-\beta \right)Y-w+\tau \right]q-\frac{p+\left(1-\beta \right)Y-s+\tau }{\eta }\int_{0}^{q}F\left(x|e\right)dx-\left(\tau T+\theta e^{2}/2\right) & q\leq F^{-1}\left(\eta |e\right)\\ \left[p+\left(1-\beta \right)Y-s+\tau \right]F^{-1}\left(\eta |e\right)-\left(w-s\right)q-\frac{p+\left(1-\beta \right)Y-s+\tau }{\eta }\int_{0}^{F^{-1}\left(\eta |e\right)}F\left(x|e\right)dx-\left(\tau T+\theta e^{2}/2\right) & q>F^{-1}\left(\eta |e\right) \end{array}$$ (5.4)

**Proof.** Refer to Appendix A6.

Theorem 5.1 explicitly provides the expression of the risk-averse NEV retailer’s CVaR under the SS-SRP contract. With all variables given, if the retailer’s ordering quantity is *q* and the sales effort level is *e*, the CVaR the retailer achieves, denoted as *CVaR*(*π*_*r*_(*q*, *e*)), can be calculated using Eq (5.4). Considering problem (5.2), the risk-averse retailer intends to determine the optimal order quantity and sales effort level with the objective of maximizing *CVaR*(*π*_*r*_(*q*, *e*)). According to the CVaR expression provided by Theorem 5.1, we can obtain the retailer’s optimal decisions under the SS-SRP contract, shown in the following proposition.

**Proposition 5.1.** *Under the SS-SRP contract*, *the optimal joint decision of the risk-averse retailer is* (q*, e*), *where*

$$q^{*}=\Phi^{-1}\left(\frac{\eta \left[p+\left(1-\beta \right)Y-w+\tau \right]}{p+\left(1-\beta \right)Y-s+\tau }\right)+z\left(e^{*}\right)$$ (5.5)

$$\left[p+\left(1-\beta \right)Y-w+\tau \right]z\prime \left(e^{*}\right)-\theta e^{*}=0$$ (5.6)

**Proof.** Refer to Appendix A7.

Proposition 5.1 provides the risk-averse NEV retailer’s joint decision on the order quantity and sales effort level (q*, e*) under the SS-SRP contract. With an adequate consideration of the risk arising from the demand uncertainty, (q*, e*) is obtained with the objective of maximizing CVaR measurement. When the retailer’s confidence level and other parameters are given, the unique optimal order quantity and sales effort level can be calculated by Eqs (5.5) and (5.6).

Similar to Proposition 3.1, we can easily prove that both q* and e* increase with respect to the government subsidies (proof omitted). This means that in the decentralized case, the increase of government subsidies can induce the retailer to make more sales efforts and order more NEVs. However, the retailer’s strategy is aimed at obtaining more subsidies from the government, so the government subsidies may become the dependence for the retailer. Therefore, the promotion effect of government subsidies may be accompanied by a negative influence on the sustainable development of the NEV supply chain.

Besides the government subsidies, the effect of the retailer’s risk attitude is another point we are interested in. By analyzing the relationship between the retailer’s decisions and the confidence level, we can find how the risk aversion degree influences the retailer’s optimal order quantity and sales effort level.

**Proposition 5.2.** *Under the SS-SRP contract*, *the retailer’s optimal order quantity increases with respect to the confidence level*, *while the optimal sales effort level is not affected by the confidence level*.

**Proof.** Refer to Appendix A8.

A lower confidence level indicates a higher risk aversion degree. Thus, Proposition 5.2 shows that an increase of the risk aversion degree will reduce the retailer’s order quantity but has no influence on his sales effort decision. Facing the demand uncertainty, a more risk-averse retailer will be more conservative and order less NEVs, which is consistent with the conclusions of previous research. On the other hand, no matter what the retailer’s risk attitude is, more sales efforts can always enhance market demand and promote sales. So the decision on sales effort level only depends on the balance between the profit generated and the incurred cost, and has no relationship with the risk aversion degree.

Since *τ* acts as a rebate or a penalty when the realized sales quantity is more or less than the sales target *T*, the NEV retailer will certainly try to obtain more rebates rather than being punished. We can see in Proposition 5.1 that the retailer’s decisions regarding q* and e* are dependent on *τ* with other variables given. The following proposition reveals how the unit rebate/penalty influences the retailer’s decisions.

**Proposition 5.3.** *Under the SS-SRP contract*, *the NEV retailer’s optimal order quantity and sales effort level both increase with respect to the unit rebate/penalty*.

**Proof.** Refer to Appendix A9.

Proposition 5.3 indicates that an increase of the unit rebate/penalty in the contract will induce the NEV retailer to make more sales efforts and result in a larger order quantity. Although *τ* may act as a penalty and is negative for the retailer, the retailer has the initiative to improve the sales effort level attempting to sell more NEVs than the sales target *T*, and thus can avoid being punished and acquire more rebates from the manufacturer. It is worth mentioning that *T* has no effect on the retailer’s decisions and just affects the profit allocation between the manufacturer and the retailer.

### 5.2 The SS-SRP coordination contract design

With the purpose of coordinating the NEV supply chain, the manufacturer should offer the retailer a well-designed SS-SRP contract. The optimal centralized solutions shown in Theorem 3.1 provide guidelines for determining the SS-SRP contract parameters to achieve coordination. In order to maximize the supply chain’s profit, the offered SS-SRP contract should induce the retailer to make individual decisions aligning with the optimal centralized solutions of the integrated supply chain. Thus the coordination parameters can be derived by letting the risk-averse retailer’s CVaR-maximizing sales effort level and order quantity equal to those of the integrated supply chain, i.e., $e^{*}=e_{s}^{*}$ and $q^{*}=q_{s}^{*}$. Comparing Eq (3.2) with Eq (5.5) and Eq (3.3) with Eq (5.6), we can determine appropriate parameters of the SS-SRP contract to coordinate the NEV supply chain, shown in the following theorem.

**Theorem 5.2.** *The SS-SRP coordination contract parameters for the NEV supply chain are presented as follows*:

w=c−(1−η)(p+Y−s) (5.7)

τ=βY−(1−η)(p+Y−s) (5.8)

**Proof.** Refer to Appendix A10.

Theorem 5.2 provides an SS-SRP contract to achieve the NEV supply chain coordination. The coordination contract shows the values of the wholesale price and the unit rebate/penalty. When all other related parameters are given, the values of *w* and *τ* calculated according to Theorem 5.2 can lead the risk-averse retailer to make individual decisions equaling to the integrated supply chain’s profit-maximizing solutions. There are several special cases of the SS-SRP coordination contract that need to be further discussed.

For example, when *τ* = 0, the SS-SRP contract degenerate to an SS-WP contract that cannot achieve coordination. Therefore, *τ* should be positive and there should be *β* > (1 − *η*) (*p* + *Y* − *s*)/*Y*. Further, we have *w* + *βY* − *c* > 0, which results in a positive profit for the manufacturer. Thus, the manufacturer will have the aspiration to participate in the business.

As mentioned previously, *β* = 1 is a special case in which the manufacturer occupies all the government subsidies rather than sharing them with the retailer. Actually, when *β* = 1, the SS-SRP contract degenerate to a single SRP contract. From Theorem 5.2, although the NEV supply chain can still be coordinated when *β* = 1, Eqs (5.5) and (5.6) show that the SS policy can induce the retailer to make more sales efforts and order more NEVs (proof similar to that of Proposition 3.1 is omitted here). In fact, *τ* = *βY* − (1 − *η*) (*p* + *Y* − *s*) shows that *τ* is increased when *β* = 1. It means that if the manufacturer refuses to share the government subsidies with the retailer, *τ* should be enlarged to induce the retailer to make more sales efforts and order more NEVs. Therefore, we can consider the SS policy as a supplementary for achieving coordination.

When *η* = 1, the NEV retailer becomes risk-neutral and the SS-SRP coordination contract parameters are *w* = *c* and *τ* = *βY*. Facing a risk-neutral retailer, in order to maximize the NEV supply chain’s total profit, the manufacturer should offer an SS-SRP contract in which the wholesale price is equal to the production cost and the government subsidies shared by the manufacturer are completely used to pay the rebate to the retailer. The manufacturer can obtain profit by adjusting the sales target *T*. Facing a risk-averse retailer, the parameters *w* and *τ* in the SS-SRP coordination contract are both modified by −(1 − *η*)(*p* + *Y* − *s*). To induce the risk-averse retailer to make decisions equaling to the centralized decisions, the manufacturer cuts the wholesale price by −(1 − *η*)(*p* + *Y* − *s*). Simultaneously, the unit rebate/penalty is reduced by a same amount, so that the manufacturer reserves a part of the shared government subsidies and the reserved part is (1 − *η*)(*p* + *Y* − *s*). By reducing *τ*, the NEV manufacturer remedies her concession on the wholesale price.

When *Y = 0*, the NEV supply chain can be considered as an ICEV case and the corresponding contract parameters are *w* = *c* − (1 − *η*)(*p* − *s*) and *τ* = − (1 − *η*)(*p* − *s*) < 0. In such a situation, obviously the manufacturer cannot obtain any profit and the ICEV supply chain cannot be coordinated by the SRP contract. However, for the NEV supply chain, although *w* − *c =* − (1 − *η*)(*p* − *s*)≤ 0, the manufacturer can obtain positive profit by sharing the government subsidies. On the other hand, the NEV retailer can promote sales by making sales effort and further obtain rebates from the manufacturer. That is to say, a well-designed SS-SRP contract can maximize the NEV supply chain’s total profit and meanwhile guarantee the profits of both the manufacturer and the retailer. Thus, the NEV supply chain can be coordinated and maintained sustainably.

By involving the confidence level *η*, the proposed SS-SRP contract considers the retailer’s risk attitude. Another attractive issue is the effect of the retailer’s risk aversion degree on the contract parameters. Eqs (5.7) and (5.8) show that both the wholesale price *w* and the unit rebate/penalty *τ* are related to *η*.

**Proposition 5.4.** *In the SS-SRP coordination contract*, *both the wholesale price and the unit rebate/penalty increase with respect to the retailer’s confidence level*.

**Proof.** Refer to Appendix A11.

According to Proposition 5.4, in order to achieve coordination, both the wholesale price and the unit rebate/penalty in the SS-SRP contract should be decreased when the NEV retailer is more risk-averse. A more risk-averse NEV retailer tends to be more conservative and order less. A lower wholesale price can induce the retailer to enlarge the order quantity (easily obtained by observing Eqs (5.5) and (5.6) and the proof is omitted) so that the NEV supply chain’s total profit can be optimized. Suffering from a lower wholesale price, the manufacturer remedies her concession by decreasing the unit rebate paid to the retailer. Thus, the profits of both the manufacturer and the retailer can be guaranteed and the NEV supply chain can be coordinated and maintained sustainably.

### 5.3 Coordination efficiency evaluation

The combined SS-SRP contract characterized in Theorem 5.2 is proved able to coordinate the NEV supply chain. In this subsection, we evaluate the coordination efficiency of such a contract and testify its superiority by comparing it to the SS-WP contract (an example of non-coordination contracts).

According to the discussions about Proposition 4.2, although the SS-WP contract cannot coordinate the NEV supply chain, the manufacturer and retailer still may participate in the business under the SS-WP contract when the parameters satisfy the conditions *w*_*w*_ + *β*_*w*_*Y* − *c* >0 and *p* + (1 − *β*_*w*_)*Y* − *w*_*w*_ > 0. By choosing appropriate parameters according to Theorem 5.2, the proposed SS-SRP contract can successfully align the decisions of the risk-averse NEV retailer with the centralized decisions of the integrated NEV supply chain. We indicate the superiority of the SS-SRP coordination contract by comparing it to the SS-WP contract and summarize the corresponding conclusion in the following proposition.

**Proposition 5.5.** *With the SS-SRP coordination contract, the risk-averse NEV retailer’s decisions on order quantity and sales effort level dominate over those under the non-coordinating SS-WP contract, i.e.*, $q^{*}>q_{w}^{*}$, $e^{*}>e_{w}^{*}$.

**Proof.** Refer to Appendix A12.

Proposition 5.5 indicates that the risk-averse NEV retailer’s decisions on order quantity and sales effort level are enhanced under the SS-SRP coordination contract compared to the non-coordination SS-WP contract. This means that the proposed SS-SRP contract can successfully promote the NEV sales. In order to testify whether the profits of the NEV retailer and manufacturer are higher under the SS-SRP coordination contract, we evaluate the profit allocation of the NEV supply chain. We provide the following proposition to show the performance of the SS-SRP contract in allocating the supply chain profit.

**Proposition 5.6.** *Under the SS-SRP coordination contract*, *an arbitrary allocation of the supply chain profit can be achieved by varying the sales target T*.

**Proof.** Refer to Appendix A13.

With $q_{s}^{*}$ and $e_{s}^{*}$ determined, the size of *T* in the coordination contract decides the profit allocation between the NEV retailer and manufacturer. Moreover, Eq (A.3)/Eq (A.4) shows that the expected profit of the retailer/manufacturer increases/decreases linearly with respect to *T*. Although the manufacturer can determine *T* according to the negotiating power contrast with the retailer, she should set an attractive *T* to ensure the retailer earn more than the non-coordination case so that the retailer has the aspiration to accept the offered contract and participate in the business. Cachon (2003) [24] indicates that a contract arbitrarily allocating the supply chain profit can lead to a coordination situation under which at least one player’s profit is enhanced without reducing other players’ profits. In other words, a Pareto-improving win-win situation can be achieved. According to Proposition 5.6, a well-designed SS-SRP contract can arbitrarily allocate the supply chain profit and result in a Pareto-improving win-win situation compared to the non-coordination case. Combining Proposition 5.5 with Proposition 5.6, the proposed SS-SRP contract with appropriate parameters can promote the NEV sales and meanwhile lead to a Pareto improvement. We can conclude that the SS-SRP contract is effective in coordinating the NEV supply chain.

By incorporating the confidence level *η*, the proposed SS-SRP contract considers the effect of the NEV retailer’s risk aversion. Proposition 5.2 and Proposition 5.4 demonstrate that the retailer’s risk aversion degree affects the retailer’s decisions as well as the contract parameters to some extent. To understand how risk aversion affects the supply chain’s profit allocation, the following proposition is put forth.

**Proposition 5.7.** *Under the SS-SRP coordination contract*, *as the NEV retailer’s confidence level increases*, *the retailer’s expected profit decreases and the manufacturer’s expected profit increases*.

**Proof.** Refer to Appendix A14.

Proposition 5.7 indicates that a more risk-averse retailer will occupy more profit in the NEV supply chain. The manufacturer’s profit will be reduced when collaborating with a more risk-averse retailer. Recall the explanation of Theorem 5.2, as a response to the effect of the retailer’s risk aversion, the wholesale price *w* and the unit rebate/penalty *τ* in the SS-SRP contract are both decreased by the same amount, −(1 − *η*)(*p* + *Y* − *s*). The decrease of *w* is to induce the retailer to make more sales efforts and order more NEVs to align with the optimal centralized solutions of the integrated supply chain. The decrease of *τ* is to reduce the rebates the manufacturer pays to the retailer and protect the manufacturer’s profit. However, since the decrease of *w* is for the whole order quantity and the decrease of *τ* is only for the part of the order quantity exceeding the sales target, decreasing *τ* by the same amount cannot completely compensate the manufacturer’s concession on the wholesale price. Consequently, the manufacturer’s profit is reduced by the retailer’s risk aversion. For the retailer, although the unit rebate is reduced by his risk aversion, the wholesale price is reduced by the same amount for more NEVs, the retailer will obtain a higher profit as a result. In conclusion, under the SS-SRP coordination contract, the retailer benefits from his risk aversion while the manufacturer suffers from the retailer’s risk aversion. But if the retailer’s risk aversion degree is extremely high, the manufacturer will face losses and quit the business. In other words, a NEV manufacturer is not willing to collaborate with a highly risk-averse NEV retailer.

Under the SS-SRP coordination contract, the NEV retailer’s sales effort $e_{s}^{*}$ increases the sales and further enhances the supply chain profit. However, Eqs (A.3) and (A.4) indicate that the sales effort only affects the retailer’s profit and has no effect on the manufacturer’s profit. The conclusion is summarized in the following proposition.

**Proposition 5.8.** *Under the SS-SRP coordination contract*, *the NEV retailer occupies all the profit increase generated by the sales effort and there is no free riding phenomenon for the manufacturer*.

**Proof.** Refer to Appendix A15.

In Eqs (A.6) and (A.8), the first item $\left(p+Y-c\right)z\left(e_{s}^{*}\right)$ represents the income generated by the sales effort and the second item $-\theta e_{s}^{*2}/2$ stands for the incurred cost. From Proposition 5.8, under the SS-SRP coordination contract, the retailer’s sales effort cost is fully paid off and the manufacturer does not obtain any additional profit caused by the sales effort. The retailer has the initiative to make more sales efforts, order and sell more NEVs, and further obtain more rebates from the manufacturer. The sales effort will make the retailer have all the generated profit increase. Even though the manufacturer does not benefit directly from the retailer’s sales effort, the sales effort should certainly be encouraged since it can promote the NEV order quantity as well as enhance the supply chain profit. That is beneficial for the development of the NEV commerce. Moreover, the NEV retailer may make a concession on the sales target when he obtains a high profit and further indirectly enhance the manufacturer’s profit.

## 6. Numerical experiments

So far we have proposed a combined SS-SRP contract, derived the contract parameters to successfully coordinate the NEV supply chain, and evaluated the coordination efficiency. In order to further illustrate the properties of the proposed model, we carry out numerical experiments to explore how some parameters affect the order quantities and the profit allocation in the NEV supply chain under the SS-WP non-coordination contract and the SS-SRP coordination contract. The comparison between different contracts demonstrates the superiority of the proposed SS-SRP contract and provides guidelines for sustainably maintaining the NEV supply chain by balancing the profit allocation between the NEV retailer and manufacturer.

In the additive form of market demand *x* = *z*(*e*) + *ξ*, we assume that *z*(*e*) = *e* and the stochastic part *ξ* is subject to a normal distribution whose mean value is *μ* = 1000 and standard deviation is *σ*. Other parameters are assumed as follows: the per unit retail price *p* = 80000, the per unit production cost *c* = 100000, the per unit salvage value *s* = 40000, the manufacturer’s SS proportion *β* = 0.8, and sales effort cost coefficient *θ* = 100.

**Fig 2:**
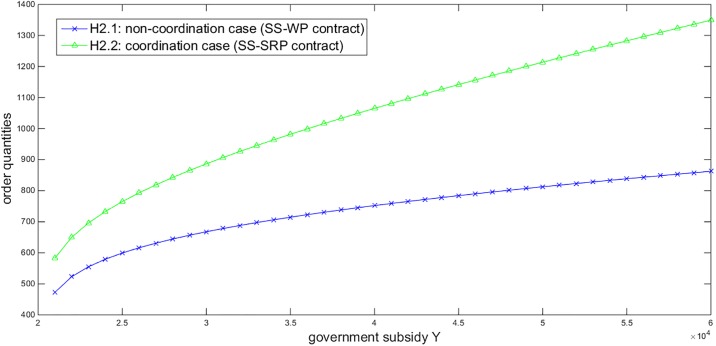
Impact of *Y* on the order quantities (*η =* 0.9, *σ* = 200).

**Fig 3:**
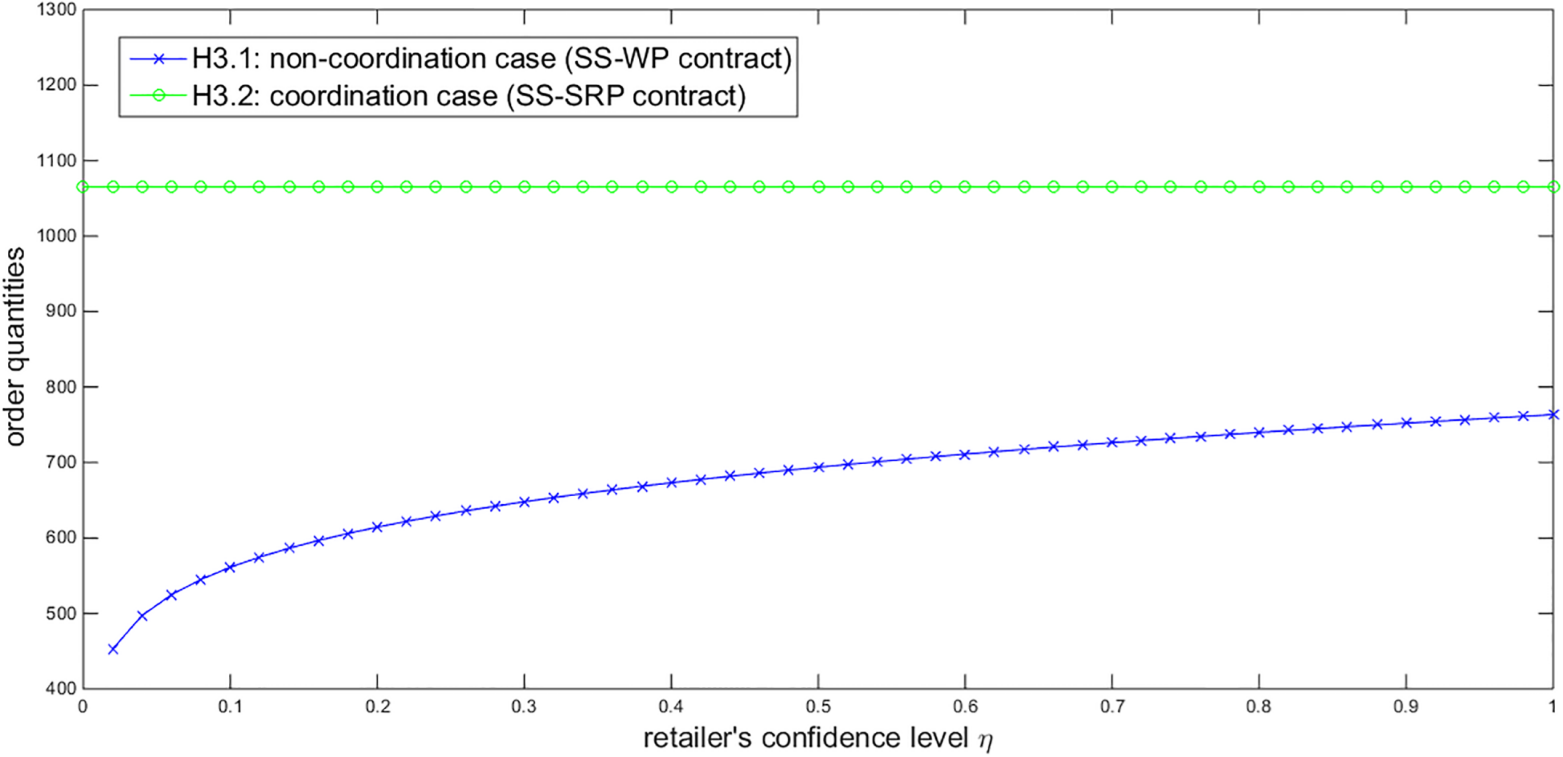
Impact of *η* on the order quantities (*Y* = 40000, *σ* = 200).

**Fig 4:**
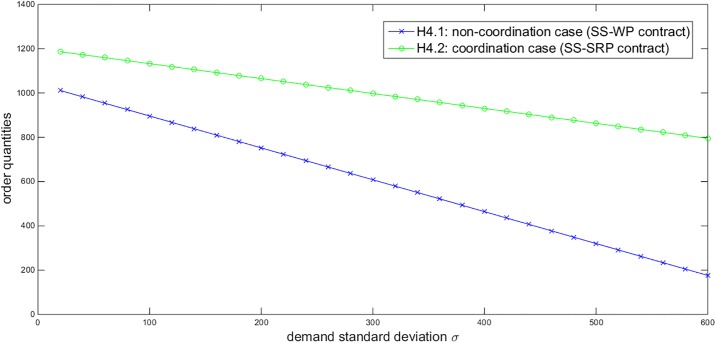
Impact of *σ* on the order quantities (*Y* = 40000, *η =* 0.9).

Based on the above numerical assumptions, we first illustrate the effects of some parameters on the order quantities under the SS-WP and SS-SRP contracts. Given *η* = 0.9 and *σ* = 200, Fig 2 describes that the order quantities under both contracts increase in the government subsidies *Y*. Given *Y* = 40000 and *σ* = 200, Fig 3 displays that the order quantity under the SS-WP contract increases in the retailer’s confidence level *η* while the order quantity under the SS-SRP coordination contract is not affected by *η*. Given *Y* = 40000 and *η* = 0.9, Fig 4 shows that the order quantities under both contracts decrease in the demand standard deviation *σ*. These three figures all demonstrate that the order quantity under the SS-SRP coordination contract is always larger than that under the SS-WP contract. Thus it is verified that the proposed SS-SRP contract can promote the NEV sales. Moreover, we can also observe that the promoting effect of the SS-SRP contract compared to the SS-WP contract is enhanced when the government subsidies increase, the retailer becomes more risk-averse, or the demand uncertainty enlarges.

**Fig 5:**
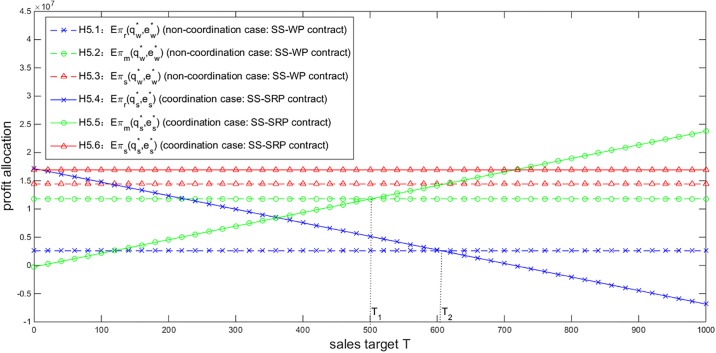
Impact of *T* on the profit allocation (*Y* = 40000, *η =* 0.9, *σ* = 200).

Next we illustrate the effect of the sales target *T* on the profit allocation under the SS-SRP contract to explore how to determine *T* appropriately. As the comparison object, the profit allocation under the SS-WP contract, which has nothing to do with *T*, is also involved. Given *Y* = 40000, *η* = 0.9 and *σ* = 200, Fig 5 describes that with the increase of *T*, the NEV retailer’s profit is reduced while the NEV manufacturer’s profit is enlarged, and the total profit of the supply chain stays invariant. By setting a higher *T* to avoid paying a large amount of rebates to the retailer for the exceeding sales quantity, the manufacturer can occupy more profit in the supply chain. However, if *T* exceeds a certain level *T*_2_ (marked in Fig 5), the retailer will obtain a lower profit under the SS-SRP contract than under the SS-WP contract, or face a loss. The retailer therefore prefers the SS-WP contract or quits the business. On the other hand, if *T* is lower than another certain level *T*_1_ (also marked in Fig 5), the manufacturer will obtain a lower profit under the SS-SRP contract than under the SS-WP contract, or face a loss. The manufacturer therefore prefers the SS-WP contract or has no aspiration to participate in the business. In other words, *T* should be appropriately chosen in the scope (*T*_1,_ *T*_2_) to ensure both players obtain higher profits under the SS-SRP contract than under the SS-WP contract and accept the SS-SRP contract. The specific value of T in the contract, which directly decides the profit allocation in the NEV supply chain, may be determined by the negotiating power contrast between the manufacturer and the retailer.

Combining the analysis about Fig 5 with the coordination contract parameters shown in Theorem 5.2 and the relevant discussions, we provide the following observation to further explain the principles of designing the SS-SRP contract.

**Observation 6.1.** *In order to coordinate and sustainably maintain the NEV supply chain*, *the four parameters in the SS-SRP contract* (*w*, *τ*, *β*, *T*) *should satisfy the following conditions*:

*(a) w* = *c* − (1 − *η*)(*p* + *Y* − *s*)*;*
*(b) τ* = *βY* − (1 − *η*)(*p* + *Y* − *s*)*;*
*(c) β >* (1 − *η*)(*p* + *Y* − *s*)/*Y;*
*(d) T* ∈ (*T*_1,_*T*_2_).

Among the four necessary conditions presented in Observation 6.1, (a) and (b) are set to align the risk-averse NEV retailer’s decisions with the centralized decisions and thus maximize the supply chain’s total profit; (c) is set to satisfy *w* + *βY* − *c* > 0 and thus guarantee that the manufacturer can obtain profit from each NEV ordered by the retailer; and (d) is set to guarantee both players can obtain higher profits under the SS-SRP contract than under the SS-WP contract and are willing to accept the SS-SRP contract. These four conditions are necessary for coordinating and sustainably maintaining the NEV supply chain. If any one of them is not satisfied, the NEV supply chain cannot be optimized or one player will reject the SS-SRP contract.

**Fig 6:**
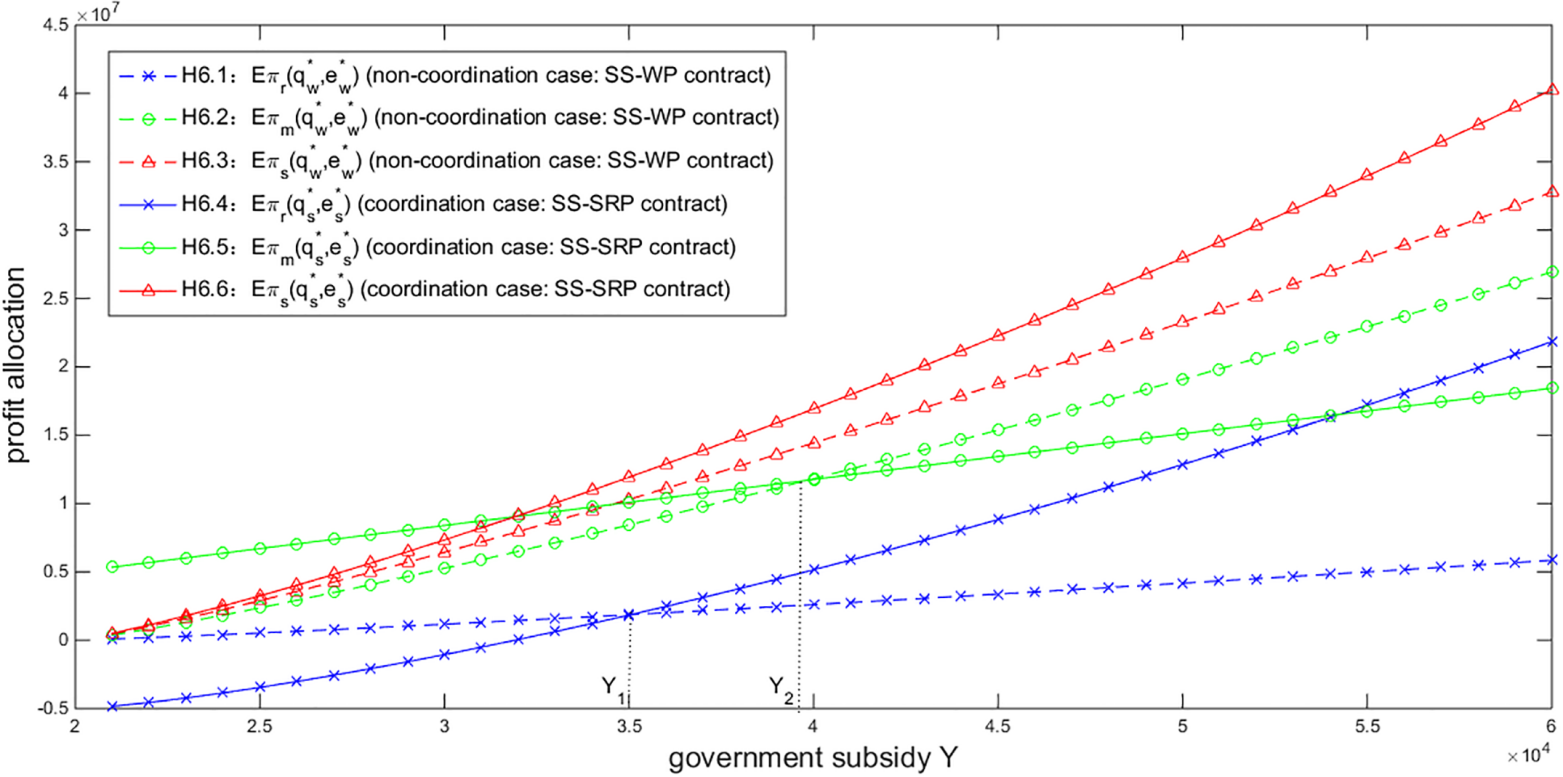
Impact of *Y* on the profit allocation (*T* = 500, *η* = 0.9, *σ* = 200).

As a major characteristic of the NEV industry, the government subsidies play an important role in the profit allocation of the NEV supply chain. Given *T* = 500, *η* = 0.9 and *σ* = 200, Fig 6 shows the impact of *Y* on the profit allocation under the SS-WP contract and the SS-SRP coordination contract. We can see that an increase of *Y* enlarges the profits of the retailer, the manufacturer and the entire supply chain under either contract. As a profit source outside the NEV supply chain, the government subsidies benefit both players no matter which contract is adopted. However, if *Y* is lower than a certain level *Y*_1_ (marked in Fig 6), the retailer will obtain a lower profit under the SS-SRP contract than under the SS-WP contract, or face a loss. The retailer therefore prefers the SS-WP contract or has no aspiration to participate in the business. On the other hand, if *Y* exceeds another certain level *Y*_2_ (also marked in Fig 6), the manufacturer will obtain a lower profit under the SS-SRP contract than under the SS-WP contract and thus prefer the SS-WP contract. In other words, *Y* should be appropriately chosen in the scope (*Y*_1_, *Y*_2_) to ensure both players obtain higher profits under the SS-SRP contract than under the SS-WP contract and accept the SS-SRP contract. We summarize such an observation as follows to offer a suggestion for the government to determine the subsidies.

**Observation 6.2.** *In order to coordinate and sustainably maintain the NEV supply chain*, *the government subsidies should be set in the scope* (*Y*_1_, *Y*_2_).

In addition, by observing Fig 6, we can find the NEV retailer’s profit increases faster than the manufacturer’s with respect to *Y* under the SS-SRP coordination contract. When the government subsidies go higher, the NEV retailer tends to make more sales efforts and order more NEVs with less consideration of the effort cost because of the government’s great financial support. Thus, the retailer can obtain more rebates from the manufacturer as well as obtain more government subsidies. Therefore, the increase of government subsidies will result in a rapid growth of the retailer’s profit. On the other hand, the NEV manufacturer experiences a slow profit growth because she has to pay more rebates to the retailer with the government subsidies increasing.

**Fig 7:**
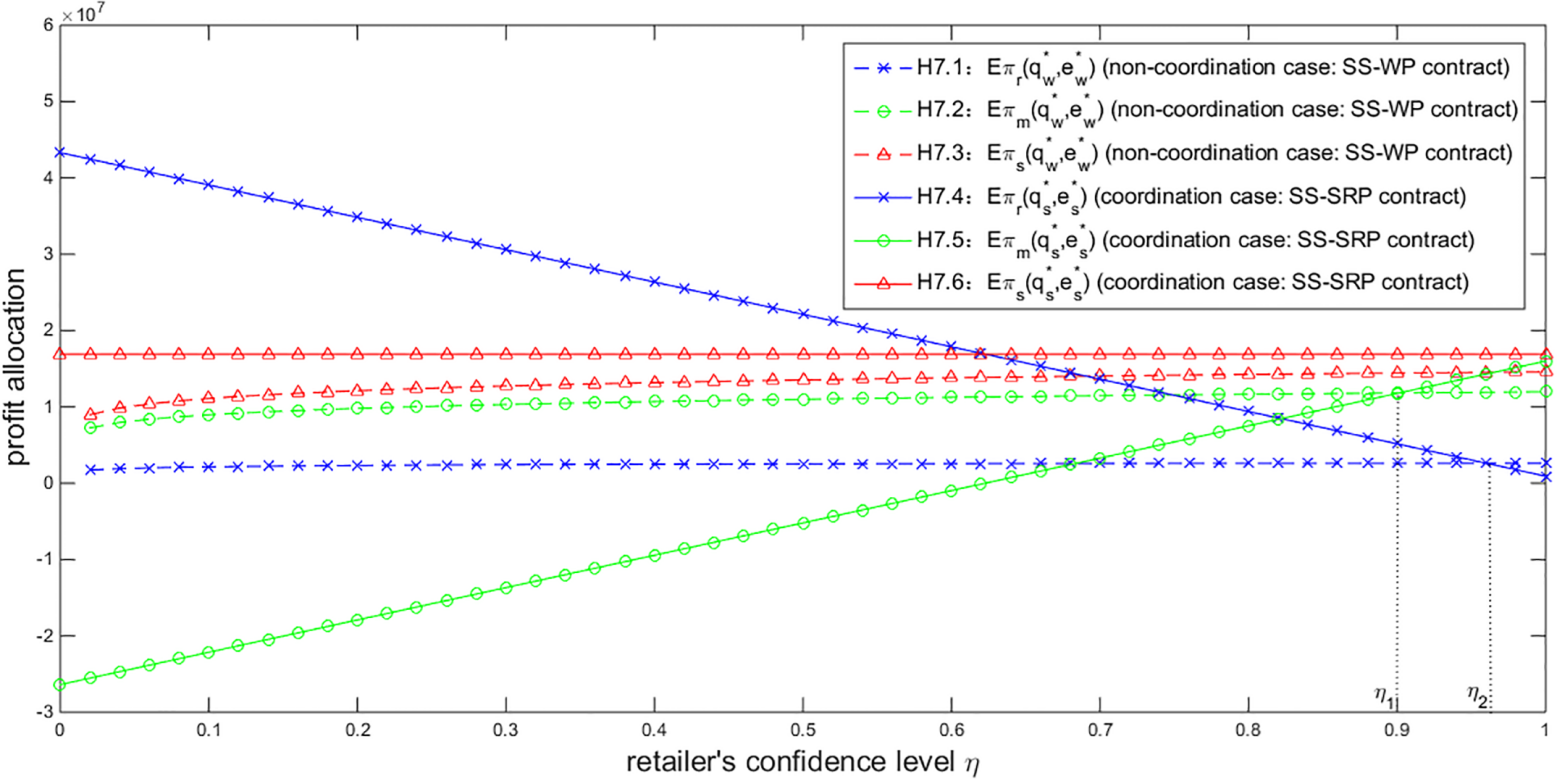
Impact of *η* on the profit allocation (*T* = 500, *Y* = 40000, *σ* = 200).

Another significant factor dramatically affecting the profit allocation is the retailer’s risk aversion degree. Given *T* = 500, *Y* = 40000 and *σ* = 200, Fig 7 characterizes the impact of *η* on the profits of the retailer, the manufacturer and the entire supply chain under different contracts. As we can see, under the SS-SRP coordination contract, an increase of *η* enlarges the manufacturer’s profit while reduces the retailer’s, and the supply chain’s total profit is not influenced by *η*. Thus, the conclusion in Proposition 5.7 is verified. Moreover, the proof of Proposition 5.7 and Fig 7 both indicate that the impact of *η* on the profit allocation under the SS-SRP coordination contract is linear. Under the SS-WP contract, an increase of *η* enlarges the profits of the retailer, the manufacturer and the entire supply chain. Under the SS-SRP coordination contract, a more risk-averse retailer will obtain more profit in the supply chain. That is to say, a lower confidence level is beneficial to the retailer. However, if *η* is lower than a certain level *η*_1_ (marked in Fig 7), the manufacturer will obtain a lower profit under the SS-SRP contract than under the SS-WP contract, or face a loss. The manufacturer therefore prefers the SS-WP contract or quits the business. The manufacturer will not collaborate with a highly risk-averse retailer under the SS-SRP contract. On the other hand, if *η* is higher than another certain level *η*_2_ (also marked in Fig 7), the retailer will obtain a lower profit under the SS-SRP contract than under the SS-WP contract and thus prefers the SS-WP contract. In other words, *η* should be appropriately set in the scope (*η*_1_, *η*_2_) to ensure both players in the NEV supply chain obtain higher profits under the SS-SRP contract than under the SS-WP contract and accept the SS-SRP contract.

**Fig 8:**
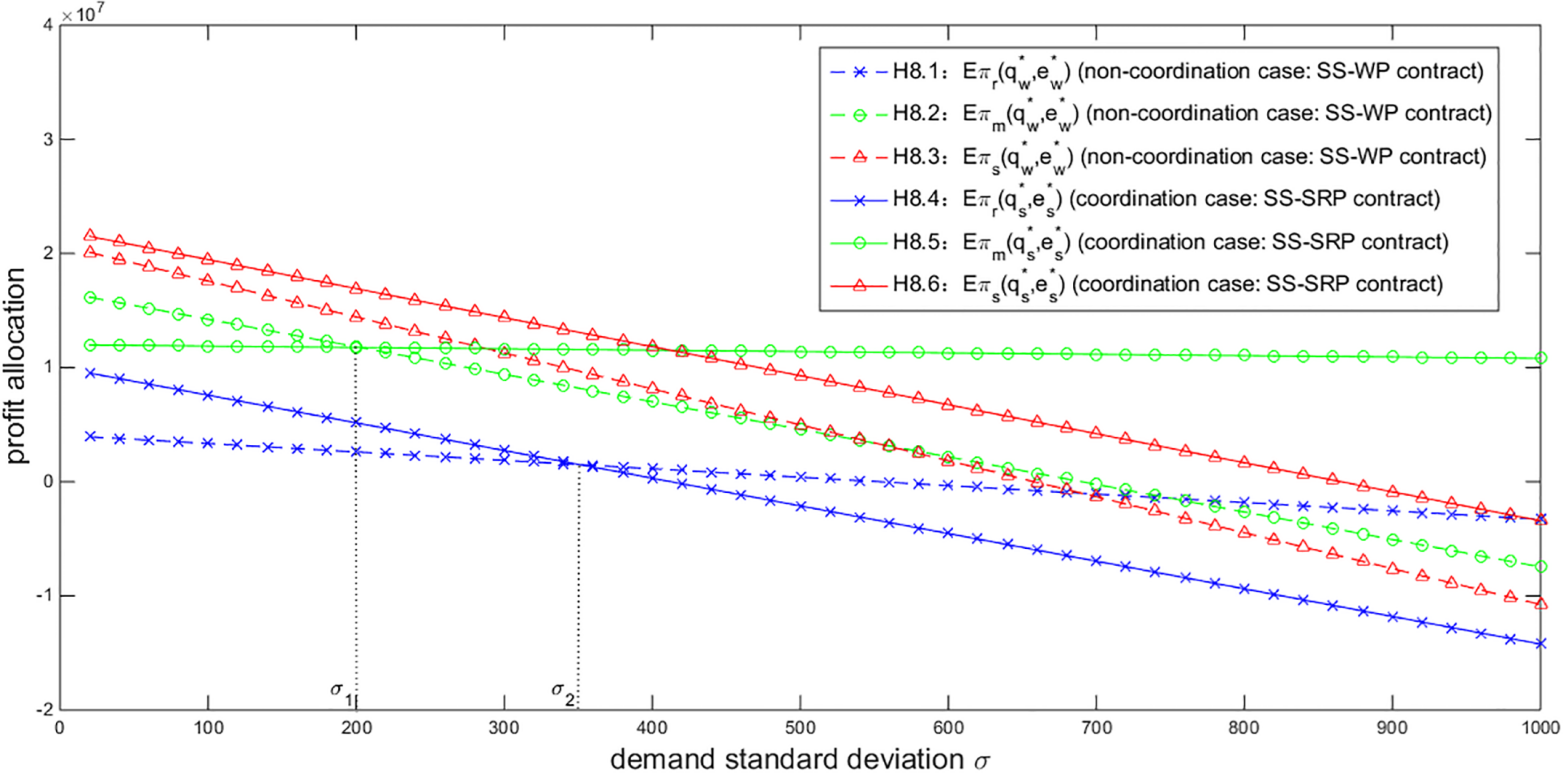
Impact of *σ* on the profit allocation (*T* = 500, *Y* = 40000, *η* = 0.9).

To address the impact of the demand uncertainty on the profit allocation, we provide another numerical experiment about the relationship between the profit allocation and the demand standard deviation *σ*. Given *T* = 500, *Y* = 40000 and *η* = 0.9, Fig 8 shows that with the increase of demand uncertainty, the profits of the retailer, the manufacturer and the entire supply chain under either the SS-WP or SS-SRP contract all decrease to different extents. No matter which contract is adopted, both players suffer from a higher demand uncertainty which brings about a greater risk for them. However, if *σ* is lower than a certain level *σ*_1_ (marked in Fig 8), the manufacturer will obtain a lower profit under the SS-SRP contract than under the SS-WP contract and thus prefers the SS-WP contract. On the other hand, if *σ* is higher than another certain level *σ*_2_ (also marked in Fig 8), the retailer will obtain a lower profit under the SS-SRP contract than under the SS-WP contract, or face a loss. The retailer therefore prefers the SS-WP contract or quits the business. In other words, only if *σ* is in the scope (*σ*_1_, *σ*_2_) will both players in the NEV supply chain obtain higher profits under the SS-SRP contract than under the SS-WP contract and accept the SS-SRP contract. Fig 8 also tells that the negative impact of the demand uncertainty on the manufacturer’s profit is reduced while that on the retailer’s is enlarged under the SS-SRP contract compared to the SS-WP contract. The SS-SRP contract transfers a part of the negative impact generated by the demand risk from the manufacturer side to the retailer side to compensate the high production cost for the NEV manufacturer.

Based on the above analysis of Figs 7 and 8, the NEV retailer’s risk aversion and the demand uncertainty are two significant factors which markedly affect the profit allocation in the NEV supply chain. By summarizing the impacts of *η* and *σ* on the profit allocation, we provide necessary conditions to ensure that both players can obtain higher profits and further sustainably maintain the NEV supply chain under the SS-SRP coordination contract.

**Observation 6.3.** *In order to coordinate and sustainably maintain the NEV supply chain*, *the retailer’s confidence level and the demand standard deviation should satisfy η* ∈ (*η*_1_,*η*_2_) *and σ* ∈ (*σ*_1_, *σ*_2_), *respectively*.

Observation 6.3 presents necessary conditions for the sustainability of a coordinated NEV supply chain. If any of the two conditions is not satisfied, the retailer or the manufacturer will quit the NEV business under the SS-SRP contract. In order to sustainably maintain the NEV supply chain, not only should the contract designer set contact parameters appropriately according to Observation 6.1 and the government offer subsidies prudently according to Observation 6.2, but also the retailer’s confidence level and the demand standard deviation should be restricted to certain scopes presented in Observation 6.3.

By observing Figs 5–8, no matter how the related parameters vary, the NEV supply chain’s total profit under the SS-SRP coordination contract is always higher than that under the SS-WP contract. There exist certain scopes for these parameters which can ensure both players obtain higher profits and accept the SS-SRP contract. Thus the effectiveness, feasibility and superiority of the proposed SS-SRP coordination contract are testified.

## 7. Concluding remarks

In this paper, we investigate the coordination and sustainability of the NEV supply chain where the risk-averse retailer makes sales effort trying to enhance the market demand. We first provide the centralized decisions of the integrated supply chain as a benchmark. In the decentralized case, supposing that the risk-averse NEV retailer makes a joint decision on the order quantity and the sales effort level with the objective of maximizing the CVaR measurement, we derive the optimal decentralized decisions under different contracts. Based on the proof that the SS-WP contract fails to coordinate the NEV supply chain, we propose a combined SS-SRP contract and design appropriate parameters to achieve coordination. It is demonstrated that a well-designed SS-SRP contract can promote the NEV sales compared to the non-coordination case and provide a Pareto-improving win-win situation by choosing appropriate contract parameters.

With model analysis and a series of numerical experiments, we focus on the impacts of several significant parameters on the profit allocation between the NEV retailer and manufacturer, and obtain the necessary conditions for sustainably maintaining the NEV supply chain. Besides the appropriate contract parameters maximizing the supply chain’s total profit, the sales target, the government subsidies, the retailer’s confidence level and the demand deviation should be in certain scopes to guarantee both players’ profits higher than non-coordination case. A profit balance between the two players can attract both of them to participate in the NEV business under the SS-SRP contract and further contribute to the sustainability of the NEV supply chain. It is also worth mentioning that under the SS-SRP coordination contract, the retailer obtains all the profit increase generated by the sales effort while the manufacturer does not benefit from the sales effort.

By incorporating the impact of risk aversion under the CVaR criterion, our model enriches the research on the NEV supply chain coordination and sustainability. The proposed coordination mechanism, which is proved to be effective, feasible and superior in promoting the NEV sales and enhancing each player’s profit, offers some guiding principles for the NEV win-win cooperation between the manufacturer and the retailer as well as a suggestion for the government to set subsidies more appropriately, which may help to promote the sustainable development of the NEV commerce.

We consider the impacts of risk aversion and government subsidies on the NEV supply chain coordination and sustainability. However, there are still many questions outstanding there. In future work, we intend to extend the model to a case in which the market demand is jointly influenced by an endogenous price and sales effort. Additionally, based on the investigation of the NEV single-channel supply chain coordination in this paper, we are also interested in how to coordinate and sustainably maintain a NEV dual-channel supply chain (traditional retail channel and online channel).

## Appendix

### A1: Proof of Theorem 3.1

Given a fixed *e*_*s*_, take the first-order derivative of *Eπ*_*s*_(*q*_*s*_, *e*_*s*_) with respect to *q*_*s*_:

$$\frac{\partial E\pi_{s}\left(q_{s},e_{s}\right)}{\partial q_{s}}=(p+Y-c)-\left(p+Y-s\right)F(q_{s}|e_{s})$$

Then taking the second-order derivative of *Eπ*_*s*_(*q*_*s*_, *e*_*s*_) with respect to *q*_*s*_, we obtain

$$\frac{\partial^{2}E\pi_{s}\left(q_{s},e_{s}\right)}{\partial q_{s}^{2}}=-\left(p+Y-s\right)f(q_{s}|e_{s})<0$$

It means that *Eπ*_*s*_(*q*_*s*_, *e*_*s*_) is concave with respect to *q*_*s*_. Letting $\frac{\partial E\pi_{s}\left(q_{s},e_{s}\right)}{\partial q_{s}}=0$, we obtain

$$F(q_{s}^{*}|e_{s})=\frac{p+Y-c}{p+Y-s}$$

As *F*(*x*|*e*_*s*_) = Φ(*x* − *z*(*e*_*s*_)), thus the supply chain’s optimal delivery quantity $q_{s}^{*}$ is obtained and shown as follows.

$$q_{s}^{*}=\Phi^{-1}\left(\frac{p+Y-c}{p+Y-s}\right)+z\left(e_{s}\right)$$

Substitute the above equality into Eq (3.1), the expected profit of the integrated supply chain can be expressed as follows.

$$\begin{array}{ll} E\pi_{s}(q_{s}^{*},e_{s}) & =\left(p+Y-c\right)q_{s}^{*}-\left(p+Y-s\right)\int_{0}^{q_{s}^{*}}F\left(x|e_{s}\right)dx-\theta e_{s}^{2}/2\\ & =\left(p+Y-c\right)\left[\Phi^{-1}\left(\frac{p+Y-c}{p+Y-s}\right)+z\left(e_{s}\right)\right]-\left(p+Y-s\right)\int_{0}^{\Phi^{-1}\left(\frac{p+Y-c}{p+Y-s}\right)+z\left(e_{s}\right)}\Phi \left(x-z\left(e_{s}\right)\right)dx-\theta e_{s}^{2}/2\\ & =\left(p+Y-c\right)\left[\Phi^{-1}\left(\frac{p+Y-c}{p+Y-s}\right)+z\left(e_{s}\right)\right]\\ & -\left(p+Y-s\right)\left[\left(\Phi^{-1}\left(\frac{p+Y-c}{p+Y-s}\right)+z\left(e_{s}\right)\right)\frac{p+Y-c}{p+Y-s}-\int_{0}^{\Phi^{-1}\left(\frac{p+Y-c}{p+Y-s}\right)+z\left(e_{s}\right)}x\phi \left(x-z\left(e_{s}\right)\right)dx\right]-\theta e_{s}^{2}/2\\ & =\left(p+Y-s\right)\int_{0}^{\Phi^{-1}\left(\frac{p+Y-c}{p+Y-s}\right)+z\left(e_{s}\right)}x\phi \left(x-z\left(e_{s}\right)\right)dx-\theta e_{s}^{2}/2\\ & =\left(p+Y-s\right)\int_{0}^{\Phi^{-1}\left(\frac{p+Y-c}{p+Y-s}\right)}\left(\xi +z\left(e_{s}\right)\right)\phi \left(\xi \right)d\xi -\theta e_{s}^{2}/2\\ & =\left(p+Y-c\right)z\left(e_{s}\right)+\left(p+Y-s\right)\int_{0}^{\Phi^{-1}\left(\frac{p+Y-c}{p+Y-s}\right)}\xi d\Phi \left(\xi \right)-\theta e_{s}^{2}/2 \end{array}$$

Take the first-order derivative of $E\pi_{s}(q_{s}^{*},e_{s})$ with respect to *e*_*s*_:

$$\frac{\partial E\pi_{s}(q_{s}^{*},e_{s})}{\partial e_{s}}=\left(p+Y-c\right)z\prime \left(e_{s}\right)-\theta e_{s}$$

Then taking the second-order derivative of $E\pi_{s}(q_{s}^{*},e_{s})$ with respect to *e*_*s*_, we obtain

$$\frac{\partial^{2}E\pi_{s}(q_{s}^{*},e_{s})}{\partial e_{s}{}^{2}}=\left(p+Y-c\right)z\prime \prime \left(e_{s}\right)-\theta <0$$

It means that $E\pi_{s}(q_{s}^{*},e_{s})$ is concave with respect to *e*_*s*_. Let $\frac{\partial E\pi_{s}(q_{s}^{*},e_{s})}{\partial e_{s}}=0$, the supply chain’s optimal sales effort level $e_{s}^{*}$ should satisfy Eq (3.3) and the corresponding optimal delivery quantity is expressed by Eq (3.2).

### A2: Proof of Proposition 3.1

According to Eq (3.3) and using the implicit function theorem, we denote

$$G_{1}\left(Y,e_{s}^{*}\right)=\left(p+Y-c\right)z\prime \left(e_{s}^{*}\right)-\theta e_{s}^{*}$$

Taking the first derivative of $e_{s}^{*}$ with respect to *Y*, we obtain

$$\frac{\partial e_{s}^{*}}{\partial Y}=-\frac{\partial G_{1}\left(Y,e_{s}^{*}\right)/\partial Y}{\partial G_{1}\left(Y,e_{s}^{*}\right)/\partial e_{s}^{*}}=-\frac{z\prime \left(e_{s}^{*}\right)}{\left(p+Y-c\right)z\prime \prime \left(e_{s}^{*}\right)-\theta }$$

As *z*(*e*) is an increasing nonconvex function with respect to *e*, we have $z\prime \left(e_{s}^{*}\right)>0$ and $z\prime \prime \left(e_{s}^{*}\right)\leq 0$. Thus $\frac{\partial e_{s}^{*}}{\partial Y}>0$, therefore $e_{s}^{*}$ increases with respect to the government subsidies.

According to Eq (3.2), taking the first derivative of $q_{s}^{*}$ with respect to *Y*, we obtain

$$\frac{\partial q_{s}^{*}}{\partial Y}=\frac{c-s}{\phi \left(\Phi^{-1}\left(\frac{p+Y-c}{p+Y-s}\right)\right){\left(p+Y-s\right)}^{2}}+z\prime \left(e_{s}^{*}\right)\frac{\partial e_{s}^{*}}{\partial Y}>0$$

Therefore $q_{s}^{*}$ increases with respect to the government subsidies.

### A3: Proof of Theorem 4.1

According to Eqs (4.1) and (4.3), the CVaR of the retailer can be expressed as:

$$\begin{array}{ll} CVaR\left(\pi_{r}\left(q_{w},e_{w}\right)\right) & =\underset{v_{w}\in R}{\text{max}}\;\left\{v_{w}-\frac{1}{\eta }E{\left[v_{w}-\pi_{r}\left(q_{w},e_{w}\right)\right]}^{+}\right\}\\ & =\underset{v_{w}\in R}{\text{max}}\;\left\{v_{w}-\frac{1}{\eta }E{\left[v_{w}-\left[p+\left(1-\beta_{w}\right)Y-w_{w}\right]q_{w}+\left[p+\left(1-\beta_{w}\right)Y-s\right]{\left(q_{w}-x\right)}^{+}+\theta e_{w}^{2}/2\right]}^{+}\right\} \end{array}$$

Denote

$$\begin{array}{ll} H\left(v_{w}\right) & =v_{w}-\frac{1}{\eta }E{\left[v_{w}-\left[p+\left(1-\beta_{w}\right)Y-w_{w}\right]q_{w}+\left[p+\left(1-\beta_{w}\right)Y-s\right]{\left(q_{w}-x\right)}^{+}+\theta e_{w}^{2}/2\right]}^{+}\\ & =v_{w}-\frac{1}{\eta }\int_{0}^{+\infty }{\left[v_{w}-\left[p+\left(1-\beta_{w}\right)Y-w_{w}\right]q_{w}+\left[p+\left(1-\beta_{w}\right)Y-s\right]{\left(q_{w}-x\right)}^{+}+\theta e_{w}^{2}/2\right]}^{+}dF\left(x|e_{w}\right)\\ & =v_{w}-\frac{1}{\eta_{r}}\int_{0}^{q_{w}}{\left[v_{w}-\left[p+\left(1-\beta_{w}\right)Y-w_{w}\right]q_{w}+\left[p+\left(1-\beta_{w}\right)Y-s\right]\left(q_{w}-x\right)+\theta e_{w}^{2}/2\right]}^{+}dF\left(x|e_{w}\right)\\ & -\frac{1}{\eta }\int_{q_{w}}^{+\infty }{\left[v_{w}-\left[p+\left(1-\beta_{w}\right)Y-w_{w}\right]q_{w}+\theta e_{w}^{2}/2\right]}^{+}dF\left(x|e_{w}\right)\\ & =v_{w}-\frac{1}{\eta }\int_{0}^{q_{w}}{\left[v_{w}+\left(w_{w}-s\right)q_{w}-\left[p+\left(1-\beta_{w}\right)Y-s\right]x+\theta e_{w}^{2}/2\right]}^{+}dF\left(x|e_{w}\right)\\ & -\frac{1}{\eta }\int_{q_{w}}^{+\infty }{\left[v_{w}-\left[p+\left(1-\beta_{w}\right)Y-w_{w}\right]q_{w}+\theta e_{w}^{2}/2\right]}^{+}dF\left(x|e_{w}\right) \end{array}$$

In order to find $v_{w}^{*}=\text{arg}\;\text{max}\;H\left(v_{w}\right)$, consider the following three cases.

1. If $v_{w}\leq -\left(w_{w}-s\right)q_{w}-\theta e_{w}^{2}/2$, then
  $$H\left(v_{w}\right)=v_{w}$$
  Take the first-order derivative of *H*(*v*_*w*_) with respect to *v*_*w*_:
  $$\frac{\partial H\left(v_{w}\right)}{\partial v_{w}}=1>0$$
  So *H*(*v*_*w*_) is increasing with respect to *v*_*w*_ when $v_{w}\leq -\left(w_{w}-s\right)q_{w}-\theta e_{w}^{2}/2$.
2. If $-\left(w_{w}-s\right)q_{w}-\theta e_{w}^{2}/2<v_{w}\leq \left[p+\left(1-\beta_{w}\right)Y-w_{w}\right]q_{w}-\theta e_{w}^{2}/2$, then
  $$\begin{array}{ll} H\left(v_{w}\right) & =v_{w}-\frac{1}{\eta }\int_{0}^{\frac{v_{w}+\left(w_{w}-s\right)q_{w}+\theta e_{w}^{2}/2}{p+\left(1-\beta_{w}\right)Y-s}}\left[v_{w}+\left(w_{w}-s\right)q_{w}-\left[p+\left(1-\beta_{w}\right)Y-s\right]x+\theta e_{w}^{2}/2\right]dF\left(x|e_{w}\right)\\ & =v_{w}-\frac{p+\left(1-\beta_{w}\right)Y-s}{\eta }\int_{0}^{\frac{v_{w}+\left(w_{w}-s\right)q_{w}+\theta e_{w}^{2}/2}{p+\left(1-\beta_{w}\right)Y-s}}F\left(x|e_{w}\right)dx \end{array}$$
  Take the first-order derivative of *H*(*v*_*w*_) with respect to *v*_*w*_:
  $$\begin{array}{ll} \frac{\partial H\left(v_{w}\right)}{\partial v_{w}} & =1-\frac{p+\left(1-\beta_{w}\right)Y-s}{\eta }\frac{1}{p+\left(1-\beta_{w}\right)Y-s}F\left(\frac{v_{w}+\left(w_{w}-s\right)q_{w}+\theta e_{w}^{2}/2}{p+\left(1-\beta_{w}\right)Y-s}|e_{w}\right)\\ & =1-\frac{1}{\eta }F\left(\frac{v_{w}+\left(w_{w}-s\right)q_{w}+\theta e_{w}^{2}/2}{p+\left(1-\beta_{w}\right)Y-s}|e_{w}\right) \end{array}$$
  Take the second-order derivative of *H*(*v*_*w*_) with respect to *v*_*w*_:
  $$\frac{\partial^{2}H\left(v_{w}\right)}{\partial v_{w}^{2}}=-\frac{1}{\eta }\frac{1}{p+\left(1-\beta_{w}\right)Y-s}f\left(\frac{v_{w}+\left(w_{w}-s\right)q_{w}+\theta e_{w}^{2}/2}{p+\left(1-\beta_{w}\right)Y-s}|e_{w}\right)<0$$
  Moreover, we have $\frac{\partial H\left(v_{w}\right)}{\partial v_{w}}|{}_{v_{w}\to -\left(w_{w}-s\right)q_{w}-\theta e_{w}^{2}/2}=1$, $\frac{\partial H\left(v_{w}\right)}{\partial v_{w}}|{}_{v_{w}\to \left[p+\left(1-\beta_{w}\right)Y-w_{w}\right]q_{w}-\theta e_{w}^{2}/2}=1-\frac{1}{\eta }F\left(q_{w}|e_{w}\right)$
3. If $v_{w}>\left[p+\left(1-\beta_{w}\right)Y-w_{w}\right]q_{w}-\theta e_{w}^{2}/2$, then
  $$\begin{array}{ll} H\left(v_{w}\right) & =v_{w}-\frac{1}{\eta }\int_{0}^{q_{w}}\left[v_{w}+\left(w_{w}-s\right)q_{w}-\left[p+\left(1-\beta_{w}\right)Y-s\right]x+\theta e_{w}^{2}/2\right]dF\left(x|e_{w}\right)\\ & -\frac{1}{\eta }\int_{q_{w}}^{+\infty }\left[v_{w}-\left[p+\left(1-\beta_{w}\right)Y-w_{w}\right]q_{w}+\theta e_{w}^{2}/2\right]dF\left(x|e_{w}\right)\\ & =v_{w}-\frac{1}{\eta }\left[\left[v_{w}-\left[p+\left(1-\beta_{w}\right)Y-w_{w}\right]q_{w}+\theta e_{w}^{2}/2\right]F\left(q_{w}|e_{w}\right)+\left[p+\left(1-\beta_{w}\right)Y-s\right]\int_{0}^{q_{w}}F\left(x|e_{w}\right)dx\right]\\ & -\frac{1}{\eta }\left[v_{w}-\left[p+\left(1-\beta_{w}\right)Y-w_{w}\right]q_{w}+\theta e_{w}^{2}/2\right]\left[1-F\left(q_{w}|e_{w}\right)\right]\\ & =v_{w}-\frac{1}{\eta }\left[p+\left(1-\beta_{w}\right)Y-s\right]\int_{0}^{q_{w}}F\left(x|e_{w}\right)dx-\frac{1}{\eta }\left[v_{w}-\left[p+\left(1-\beta_{w}\right)Y-w_{w}\right]q_{w}+\theta e_{w}^{2}/2\right] \end{array}$$
  Take the first-order derivative of *H*(*v*_*w*_) with respect to *v*_*w*_:
  $$\frac{\partial H\left(v_{w}\right)}{\partial v_{w}}=1-\frac{1}{\eta }\leq 0$$
  So *H*(*v*_*w*_) is non-decreasing with respect to *v*_*w*_ when $v_{w}>\left[p+\left(1-\beta_{w}\right)Y-w_{w}\right]q_{w}-\theta e_{w}^{2}/2$.

By analyzing the above three cases, we find that the monotonicity of *H*(*v*_*w*_) depends on the order quantity *q*_*w*_. Letting $1-\frac{1}{\eta }F\left(q_{w}|e_{w}\right)\leq 0$, we obtain that *q*_*w*_ ≥ *F*^−1^(*η*|*e*_*w*_). Thus *q*_*w*_ ∈ (0, +∞) can be partitioned into two intervals for further discussions.

1. If 0 < *q*_*w*_ ≤ *F*^−1^(*η*|*e*_*w*_), then there is $\frac{\partial H\left(v_{w}\right)}{\partial v_{w}}|{}_{v_{w}\to \left[p+\left(1-\beta_{w}\right)Y-w_{w}\right]q_{w}-\theta e_{w}^{2}/2}=1-\frac{1}{\eta }F\left(q_{w}|e_{w}\right)\geq 0$. Hence $v_{w}^{*}=\left[p+\left(1-\beta_{w}\right)Y-w_{w}\right]q_{w}-\theta e_{w}^{2}/2$, we obtain that
  $$CVaR\left(\pi_{r}\left(q_{w},e_{w}\right)\right)=H\left(v_{w}^{*}\right)=\left[p+\left(1-\beta_{w}\right)Y-w_{w}\right]q_{w}-\frac{p+\left(1-\beta_{w}\right)Y-s}{\eta }\int_{0}^{q_{w}}F\left(x|e_{w}\right)dx-\theta e_{w}^{2}/2$$
2. If *q*_*w*_ > *F*^−1^(*η*|*e*_*w*_), then there is $\frac{\partial H\left(v_{w}\right)}{\partial v_{w}}|{}_{v_{w}\to \left[p+\left(1-\beta_{w}\right)Y-w_{w}\right]q_{w}-\theta e_{w}^{2}/2}=1-\frac{1}{\eta }F\left(q_{w}|e_{w}\right)<0$. Hence $v_{w}^{*}\in \left(-\left(w_{w}-s\right)q_{w}-\theta e_{w}^{2}/2,\left[p+\left(1-\beta_{w}\right)Y-w_{w}\right]q_{w}-\theta e_{w}^{2}/2\right]$, and $v_{w}^{*}$ satisfies the first-order condition, which is $1-\frac{1}{\eta }F\left(\frac{v_{w}^{*}+\left(w_{w}-s\right)q_{w}+\theta e_{w}^{2}/2}{p+\left(1-\beta_{w}\right)Y-s}|e_{w}\right)=0$. Thus we obtain $v_{w}^{*}=\left[p+\left(1-\beta_{w}\right)Y-s\right]F^{-1}\left(\eta |e_{w}\right)-\left(w_{w}-s\right)q_{w}-\theta e_{w}^{2}/2$.
  In such case,
  $$CVaR\left(\pi_{r}\left(q_{w},e_{w}\right)\right)=H\left(v_{w}^{*}\right)=\left[p+\left(1-\beta_{w}\right)Y-s\right]F^{-1}\left(\eta |e_{w}\right)-\left(w_{w}-s\right)q_{w}-\frac{p+\left(1-\beta_{w}\right)Y-s}{\eta }\int_{0}^{F^{-1}\left(\eta |e_{w}\right)}F\left(x|e_{w}\right)dx-\theta e_{w}^{2}/2$$

### A4: Proof of Proposition 4.1

Given a fixed *e*_*w*_, according to the CVaR expression shown by Eq (4.4), consider two cases about *q*_*w*_.

When 0 < *q*_*w*_ ≤ *F*^−1^(*η*|*e*_*w*_), take the first-order derivative of *CVaR*(*π*_*r*_(*q*_*w*_, *e*_*w*_)) with respect to *q*_*w*_:

$$\frac{\partial CVaR\left(\pi_{r}\left(q_{w},e_{w}\right)\right)}{\partial q_{w}}=\left[p+\left(1-\beta_{w}\right)Y-w_{w}\right]-\frac{p+\left(1-\beta_{w}\right)Y-s}{\eta }F\left(q_{w}|e_{w}\right)$$

Then taking the second-order derivative of *CVaR*(*π*_*r*_(*q*_*w*_, *e*_*w*_)) with respect to *q*_*w*_, we obtain

$$\frac{\partial^{2}CVaR\left(\pi_{r}\left(q_{w},e_{w}\right)\right)}{\partial q_{w}^{2}}=-\frac{p+\left(1-\beta_{w}\right)Y-s}{\eta }f\left(q_{w}|e_{w}\right)<0$$

It means that *CVaR*(*π*_*r*_(*q*_*w*_, *e*_*w*_)) is concave with respect to *q*_*w*_. Letting $\frac{\partial CVaR\left(\pi_{r}\left(q_{w},e_{w}\right)\right)}{\partial q_{w}}=0$, we obtain

$$q_{w}^{*}=F^{-1}\left(\frac{\eta \left[p+\left(1-\beta_{w}\right)Y-w_{w}\right]}{p+\left(1-\beta_{w}\right)Y-s}|e_{w}\right)$$

As *F*(*x*|*e*) = Φ(*x* − *z*(*e*)), thus the supply chain’s optimal delivery quantity $q_{w}^{*}$ is obtained and shown as follows.

$$q_{w}^{*}=\Phi^{-1}\left(\frac{\eta \left[p+\left(1-\beta_{w}\right)Y-w_{w}\right]}{p+\left(1-\beta_{w}\right)Y-s}\right)+z\left(e_{w}\right)$$ (A.1)

When *q*_*w*_ > *F*^−1^(*η|e*_*w*_), taking the first-order derivative of *CVaR*(*π*_*r*_(*q*_*w*_, *e*_*w*_)) with respect to *q*_*w*_, we obtain

$$\frac{\partial CVaR\left(\pi_{r}\left(q_{w},e_{w}\right)\right)}{\partial q_{w}}=-\left(w_{w}-s\right)<0$$

So *CVaR*(*π*_*r*_(*q*_*w*_, *e*_*w*_)) is decreasing with respect to *q*_*w*_ when *q*_*w*_ > *F*^−1^(*η|e*_*w*_).

Combine the above two cases, there exists an unique optimal order quantity $q_{w}^{*}\in \left(0,F^{-1}\left(\eta |e_{w}\right)\right]$, shown by Eq (A.1).

Substitute Eq (A.1) into Eq (4.4), the corresponding CVaR expression with the optimal order quantity $q_{w}^{*}$ can be rewrite as follows.

$$\begin{array}{ll} CVaR\left(\pi_{r}\left(q_{w}^{*},e_{w}\right)\right) & =\left[p+\left(1-\beta_{w}\right)Y-w_{w}\right]q_{w}^{*}-\frac{p+\left(1-\beta_{w}\right)Y-s}{\eta }\int_{0}^{q_{w}^{*}}F\left(x|e_{w}\right)dx-\theta e_{w}^{2}/2\\ & =\left[p+\left(1-\beta_{w}\right)Y-w_{w}\right]q_{w}^{*}-\frac{p+\left(1-\beta_{w}\right)Y-s}{\eta }\int_{0}^{q_{w}^{*}}\Phi \left(x-z\left(e_{w}\right)\right)dx-\theta e_{w}^{2}/2 \end{array}$$

Take the first-order derivative of $CVaR\left(\pi_{r}\left(q_{w}^{*},e_{w}\right)\right)$ with respect to *e*_*w*_:

$$\begin{array}{ll} \frac{\partial CVaR\left(\pi_{r}\left(q_{w}^{*},e_{w}\right)\right)}{\partial e_{w}} & =\left[p+\left(1-\beta_{w}\right)Y-w_{w}\right]\frac{\partial q_{w}^{*}}{\partial e_{w}}-\frac{p+\left(1-\beta_{w}\right)Y-s}{\eta }\left[\frac{\partial q_{w}^{*}}{\partial e_{w}}\Phi \left(q_{w}^{*}-z\left(e_{w}\right)\right)-z\prime \left(e_{w}\right)\Phi \left(q_{w}^{*}-z\left(e_{w}\right)\right)\right]-\theta e_{w}\\ & =\left[p+\left(1-\beta_{w}\right)Y-w_{w}\right]\frac{\partial q_{w}^{*}}{\partial e_{w}}-\left[p+\left(1-\beta_{w}\right)Y-w_{w}\right]\left[\frac{\partial q_{w}^{*}}{\partial e_{w}}-z\prime \left(e_{w}\right)\right]-\theta e_{w}\\ & =\left[p+\left(1-\beta_{w}\right)Y-w_{w}\right]z\prime \left(e_{w}\right)-\theta e_{w} \end{array}$$

Then taking the second-order derivative of $CVaR\left(\pi_{r}\left(q_{w}^{*},e_{w}\right)\right)$ with respect to *e*_*w*_, we obtain

$$\frac{\partial^{2}CVaR\left(\pi_{r}\left(q_{w}^{*},e_{w}\right)\right)}{\partial e_{w}^{2}}=\left[p+\left(1-\beta_{w}\right)Y-w_{w}\right]z\prime \prime \left(e_{w}\right)-\theta <0$$

It means that $CVaR\left(\pi_{r}\left(q_{w}^{*},e_{w}\right)\right)$ is concave with respect to *e*_*w*_. Letting $\frac{\partial CVaR\left(\pi_{r}\left(q_{w}^{*},e_{w}\right)\right)}{\partial e_{w}}=0$, the retailer’s optimal sales effort level $e_{w}^{*}$ should satisfy Eq (4.6) and the corresponding optimal order quantity is expressed by Eq (4.5).

### A5: Proof of Proposition 4.2

As mentioned at the very beginning of Section 4, in order to maximize the supply chain profit, the SS-WP contract which can achieve coordination should ensure that the retailer makes individual decisions aligning with the optimal centralized solutions of the integrated supply chain.

Comparing Eqs (3.3) and (4.6), and letting $e_{w}^{*}=e_{s}^{*}$, we have

$$p+\left(1-\beta_{w}\right)Y-w_{w}=p+Y-c$$

Comparing Eqs (3.2) and (4.5), and letting $q_{w}^{*}=q_{s}^{*}$, we have

$$\frac{\eta \left[p+\left(1-\beta_{w}\right)Y-w_{w}\right]}{p+\left(1-\beta_{w}\right)Y-s}=\frac{p+Y-c}{p+Y-s}$$

Combining the above two equalities, we have

$$\begin{array}{ll} \{\begin{array}{l} p+\left(1-\beta_{w}\right)Y-w_{w}=p+Y-c\\ \frac{\eta \left[p+\left(1-\beta_{w}\right)Y-w_{w}\right]}{p+\left(1-\beta_{w}\right)Y-s}=\frac{p+Y-c}{p+Y-s} \end{array} & \Rightarrow \{\begin{array}{l} p+\left(1-\beta_{w}\right)Y-w_{w}=p+Y-c\\ p+\left(1-\beta_{w}\right)Y-s=\eta \left(p+Y-s\right) \end{array}\\ & \Rightarrow \{\begin{array}{l} w_{w}=c-\left(1-\eta \right)\left(p+Y-s\right)\\ \beta_{w}=\left(1-\eta \right)\frac{p+Y-s}{Y} \end{array} \end{array}$$

Only with such a pair of parameters the SS-WP contract can align the risk-averse retailer’s individual decisions with the centralized decisions of the integrated supply chain. However, under such a SS-WP contract, there is *w*_*w*_ +*β*_*w*_*Y* − *c* = 0, which indicates that the manufacturer’s profit is 0. Therefore, coordination cannot be achieved with the SS-WP contract.

### A6: Proof of Theorem 5.1

According to Eqs (5.1) and (5.3), the CVaR of the retailer can be expressed as:

$$\begin{array}{ll} CVaR\left(\pi_{r}\left(q,e\right)\right) & =\underset{v\in R}{\text{max}}\;\left\{v-\frac{1}{\eta }E{\left[v-\pi_{r}\left(q,e\right)\right]}^{+}\right\}\\ & =\underset{v\in R}{\text{max}}\;\left\{v-\frac{1}{\eta }E{\left[v-\left[p+\left(1-\beta \right)Y-w+\tau \right]q+\left[p+\left(1-\beta \right)Y-s+\tau \right]{\left(q-x\right)}^{+}+\left(\tau T+\theta e^{2}/2\right)\right]}^{+}\right\} \end{array}$$

Denote

$$\begin{array}{ll} H\left(v\right) & =v-\frac{1}{\eta }E{\left[v-\left[p+\left(1-\beta \right)Y-w+\tau \right]q+\left[p+\left(1-\beta \right)Y-s+\tau \right]{\left(q-x\right)}^{+}+\left(\tau T+\theta e^{2}/2\right)\right]}^{+}\\ & =v-\frac{1}{\eta }\int_{0}^{+\infty }{\left[v-\left[p+\left(1-\beta \right)Y-w+\tau \right]q+\left[p+\left(1-\beta \right)Y-s+\tau \right]{\left(q-x\right)}^{+}+\left(\tau T+\theta e^{2}/2\right)\right]}^{+}dF\left(x|e\right)\\ & =v-\frac{1}{\eta }\int_{0}^{q}{\left[v-\left[p+\left(1-\beta \right)Y-w+\tau \right]q+\left[p+\left(1-\beta \right)Y-s+\tau \right]\left(q-x\right)+\left(\tau T+\theta e^{2}/2\right)\right]}^{+}dF\left(x|e\right)\\ & -\frac{1}{\eta }\int_{q}^{+\infty }{\left[v-\left[p+\left(1-\beta \right)Y-w+\tau \right]q+\left(\tau T+\theta e^{2}/2\right)\right]}^{+}dF\left(x|e\right)\\ & =v-\frac{1}{\eta }\int_{0}^{q}{\left[v+\left(w-s\right)q-\left[p+\left(1-\beta \right)Y-s+\tau \right]x+\left(\tau T+\theta e^{2}/2\right)\right]}^{+}dF\left(x|e\right)\\ & -\frac{1}{\eta }\int_{q}^{+\infty }{\left[v-\left[p+\left(1-\beta \right)Y-w+\tau \right]q+\left(\tau T+\theta e^{2}/2\right)\right]}^{+}dF\left(x|e\right) \end{array}$$

In order to find v* = arg max *H*(*v*), consider the following three cases.

1. If *v* ≤ −(*w* − *s*)*q* − (*τT* + *θe*^2^/2), then
  H(v)=v
  Take the first-order derivative of *H*(*v*) with respect to *v*:
  $$\frac{\partial H\left(v\right)}{\partial v}=1>0$$
  So *H*(*v*) is increasing with respect to *v* when *v* ≤ −(*w* − *s*)*q* − (*τT* + *θe*^2^/2).
2. If −(*w* − *s*)*q* − (*τT* + *θe*^2^/2) < *v* ≤ [*p* + (1 − *β*)*Y* − *w* + *τ*]*q* − (*τT* + *θe*^2^/2), then
  $$\begin{array}{ll} H\left(v\right) & =v-\frac{1}{\eta }\int_{0}^{\frac{v+\left(w-s\right)q+(\tau T+\theta e^{2}/2)}{p+\left(1-\beta \right)Y-s+\tau }}\left[v+\left(w-s\right)q-\left[p+\left(1-\beta \right)Y-s+\tau \right]x+(\tau T+\theta e^{2}/2)\right]dF\left(x|e\right)\\ & =v-\frac{p+\left(1-\beta \right)Y-s+\tau }{\eta }\int_{0}^{\frac{v+\left(w-s\right)q+(\tau T+\theta e^{2}/2)}{p+\left(1-\beta \right)Y-s+\tau }}F\left(x|e\right)dx \end{array}$$
  Take the first-order derivative of *H*(*v*) with respect to *v*:
  $$\begin{array}{ll} \frac{\partial H\left(v\right)}{\partial v} & =1-\frac{p+\left(1-\beta \right)Y-s+\tau }{\eta }\frac{1}{p+\left(1-\beta \right)Y-s+\tau }F\left(\frac{v+\left(w-s\right)q+\left(\tau T+\theta e^{2}/2\right)}{p+\left(1-\beta \right)Y-s+\tau }|e\right)\\ & =1-\frac{1}{\eta }F\left(\frac{v+\left(w-s\right)q+\left(\tau T+\theta e^{2}/2\right)}{p+\left(1-\beta \right)Y-s+\tau }|e\right) \end{array}$$
  Take the second-order derivative of *H*(*v*) with respect to *v*:
  $$\frac{\partial^{2}H\left(v\right)}{\partial v^{2}}=-\frac{1}{\eta }\frac{1}{p+\left(1-\beta \right)Y-s+\tau }f\left(\frac{v+\left(w-s\right)q+\left(\tau T+\theta e^{2}/2\right)}{p+\left(1-\beta \right)Y-s+\tau }|e\right)<0$$
  Moreover, we have
  $$\frac{\partial H\left(v\right)}{\partial v}{|}_{v\to -\left(w-s\right)q-\left(\tau T+\theta e^{2}/2\right)}=1,\;\frac{\partial H\left(v\right)}{\partial v}{|}_{v\to \left[p+\left(1-\beta \right)Y-w+\tau \right]q-(\tau T+\theta e^{2}/2)}=1-\frac{1}{\eta }F\left(q|e\right)$$
3. If *v* > [*p* + (1 − *β*)*Y* − *w* + *τ*]*q* − (*τT* + *θe*^2^/2), then
  $$\begin{array}{ll} H\left(v\right) & =v-\frac{1}{\eta }\int_{0}^{q}\left[v+\left(w-s\right)q-\left[p+\left(1-\beta \right)Y-s+\tau \right]x+\left(\tau T+\theta e^{2}/2\right)\right]dF\left(x|e\right)\\ & -\frac{1}{\eta }\int_{q}^{+\infty }\left[v-\left[p+\left(1-\beta \right)Y-w+\tau \right]q+\left(\tau T+\theta e^{2}/2\right)\right]dF\left(x|e\right)\\ & =v-\frac{1}{\eta }\left[\left[v-\left[p+\left(1-\beta \right)Y-w+\tau \right]q+\left(\tau T+\theta e^{2}/2\right)\right]F\left(q|e\right)+\left[p+\left(1-\beta \right)Y-s+\tau \right]\int_{0}^{q}F\left(x|e\right)dx\right]\\ & -\frac{1}{\eta }\left[v-\left[p+\left(1-\beta \right)Y-w+\tau \right]q+\left(\tau T+\theta e^{2}/2\right)\right]\left[1-F\left(q|e\right)\right]\\ & =v-\frac{1}{\eta }\left[p+\left(1-\beta \right)Y-s+\tau \right]\int_{0}^{q}F\left(x|e\right)dx-\frac{1}{\eta }\left[v-\left[p+\left(1-\beta \right)Y-w+\tau \right]q+\left(\tau T+\theta e^{2}/2\right)\right] \end{array}$$
  Take the first-order derivative of *H*(*v*) with respect to *v*:
  $$\frac{\partial H\left(v\right)}{\partial v}=1-\frac{1}{\eta }\leq 0$$
  So *H*(*v*) is non-decreasing with respect to *v* when *v* > [*p* + (1 − *β*)*Y* − *w* + *τ*]*q* − (*τT* + *θe*^2^/2).

By analyzing the above three cases, we find that the monotonicity of *H*(*v*) depends on the order quantity *q*. Letting $1-\frac{1}{\eta }F\left(q|e\right)\leq 0$, we obtain that *q* ≥ *F*^−1^(*η|e*). Thus *q* ∈ (0,+∞) can be partitioned into two intervals for further discussions.

1. If 0 < *q* ≤ *F*^−1^(*η|e*), then there is $\frac{\partial H\left(v\right)}{\partial v}|{}_{v\to \left[p+\left(1-\beta \right)Y-w+\tau \right]q-\left(\tau T+\theta e^{2}/2\right)}=1-\frac{1}{\eta }F\left(q|e\right)\geq 0$. Hence v* > [*p* + (1 − *β*)*Y* − *w* + *τ*]*q* − (*τT* + *θe*^2^/2), we obtain that
  $$CVaR\left(\pi_{r}\left(q,e\right)\right)=H\left(v^{*}\right)=\left[p+\left(1-\beta \right)Y-w+\tau \right]q-\frac{p+\left(1-\beta \right)Y-s+\tau }{\eta }\int_{0}^{q}F\left(x|e\right)dx-\left(\tau T+\theta e^{2}/2\right)$$
2. If *q* > *F*^−1^(*η|e*), then there is $\frac{\partial H\left(v\right)}{\partial v}|{}_{v\to \left[p+\left(1-\beta \right)Y-w+\tau \right]q-\left(\tau T+\theta e^{2}/2\right)}=1-\frac{1}{\eta }F\left(q|e\right)<0$. Hence v* (∈ −(*w* − *s*)*q* − (*τT* + *θe*^2^/2), [*p* + (1 − *β*)*Y* − *w* + *τ*]*q* − (*τT* + *θe*^2^/2)], and v* satisfies the first-order condition, which is $1-\frac{1}{\eta }F\left(\frac{v^{*}+\left(w-s\right)q+\left(\tau T+\theta e^{2}/2\right)}{p+\left(1-\beta \right)Y-s+\tau }|e\right)=0$. Thus we obtain v* = [*p* + (1 − *β*)*Y* − *s* + *τ*]*F*^−1^(*η|e*)−(*w* − *s*)*q*−(*τT* + *θe*^2^/2).
  In such case,
  $$CVaR\left(\pi_{r}\left(q,e\right)\right)=H\left(v^{*}\right)=\left[p+\left(1-\beta \right)Y-s+\tau \right]F^{-1}\left(\eta |e\right)-\left(w-s\right)q-\frac{p+\left(1-\beta \right)Y-s+\tau }{\eta }\int_{0}^{F^{-1}\left(\eta |e\right)}F\left(x|e\right)dx-\left(\tau T+\theta e^{2}/2\right)$$

### A7: Proof of Proposition 5.1

Given a fixed *e*, according to the CVaR expression shown by Eq (5.4), consider two cases about *q*.

When 0 < *q* ≤ *F*^−1^(*η|e*), take the first-order derivative of *CVaR*(*π*_*r*_(*q*, *e*)) with respect to *q*:

$$\frac{\partial CVaR\left(\pi_{r}\left(q,e\right)\right)}{\partial q}=\left[p+\left(1-\beta \right)Y-w+\tau \right]-\frac{p+\left(1-\beta \right)Y-s+\tau }{\eta }F\left(q|e\right)$$

Then taking the second-order derivative of *CVaR*(*π*_*r*_(*q*, *e*)) with respect to *q*, we obtain

$$\frac{\partial^{2}CVaR\left(\pi_{r}\left(q,e\right)\right)}{\partial q^{2}}=-\frac{p+\left(1-\beta \right)Y-s+\tau }{\eta }f\left(q|e\right)<0$$

It means that *CVaR*(*π*_*r*_(*q*, *e*)) is concave with respect to *q*. Letting $\frac{\partial CVaR\left(\pi_{r}\left(q,e\right)\right)}{\partial q}=0$, we obtain

$$q^{*}=F^{-1}\left(\frac{\eta \left[p+\left(1-\beta \right)Y-w+\tau \right]}{p+\left(1-\beta \right)Y-s+\tau }|e\right)$$

As *F*(*x*|*e*) = Φ(*x* − *z*(*e*)), thus the supply chain’s optimal delivery quantity q* is obtained and shown as follows.

$$q^{*}=\Phi^{-1}\left(\frac{\eta \left[p+\left(1-\beta \right)Y-w+\tau \right]}{p+\left(1-\beta \right)Y-s+\tau }\right)+z\left(e\right)$$ (A.2)

When *q* > *F*^−1^(*η|e*), taking the first-order derivative of *CVaR*(*π*_*r*_(*q*, *e*)) with respect to *q*, we obtain

$$\frac{\partial CVaR\left(\pi_{r}\left(q,e\right)\right)}{\partial q}=-\left(w-s\right)<0$$

So *CVaR*(*π*_*r*_(*q*, *e*)) is decreasing with respect to *q* when *q* > *F*^−1^(*η|e*).

Combine the above two cases, there exists an unique optimal order quantity q* ∈ (0, *F*^−1^(*η*|*e*)), shown by Eq (A.2).

Substitute Eq (A.2) into Eq (5.4), the corresponding CVaR expression with the optimal order quantity q* can be rewrite as follows.

$$\begin{array}{ll} CVaR\left(\pi_{r}\left(q^{*},e\right)\right) & =\left[p+\left(1-\beta \right)Y-w+\tau \right]q^{*}-\frac{p+\left(1-\beta \right)Y-s+\tau }{\eta }\int_{0}^{q^{*}}F\left(x|e\right)dx-\left(\tau T+\theta e^{2}/2\right)\\ & =\left[p+\left(1-\beta \right)Y-w+\tau \right]q^{*}-\frac{p+\left(1-\beta \right)Y-s+\tau }{\eta }\int_{0}^{q^{*}}\Phi \left(x-z\left(e\right)\right)dx-\left(\tau T+\theta e^{2}/2\right) \end{array}$$

Take the first-order derivative of *CVaR*(*π*_*r*_(q*, *e*)) with respect to *e*:

$$\begin{array}{ll} \frac{\partial CVaR\left(\pi_{r}\left(q^{*},e\right)\right)}{\partial e} & =\left[p+\left(1-\beta \right)Y-w+\tau \right]\frac{\partial q^{*}}{\partial e}-\frac{p+\left(1-\beta \right)Y-s+\tau }{\eta }\left[\frac{\partial q^{*}}{\partial e}\Phi \left(q^{*}-z\left(e\right)\right)-z\prime \left(e\right)\Phi \left(q^{*}-z\left(e\right)\right)\right]-\theta e\\ & =\left[p+\left(1-\beta \right)Y-w+\tau \right]\frac{\partial q^{*}}{\partial e}-\left[p+\left(1-\beta \right)Y-w+\tau \right]\left[\frac{\partial q^{*}}{\partial e}-z\prime \left(e\right)\right]-\theta e\\ & =\left[p+\left(1-\beta \right)Y-w+\tau \right]z\prime \left(e\right)-\theta e \end{array}$$

Then taking the second-order derivative of *CVaR*(*π*_*r*_(q*, *e*)) with respect to *e*, we obtain

$$\frac{\partial^{2}CVaR\left(\pi_{r}\left(q^{*},e\right)\right)}{\partial e^{2}}=\left[p+\left(1-\beta \right)Y-w+\tau \right]z\prime \prime \left(e\right)-\theta <0$$

It means that *CVaR*(*π*_*r*_(q*, *e*)) is concave with respect to *e*. Letting $\frac{\partial CVaR\left(\pi_{r}\left(q^{*},e\right)\right)}{\partial e}=0$, the retailer’s optimal sales effort level e* should satisfy Eq (5.6) and the corresponding optimal order quantity is expressed by Eq (5.5).

### A8: Proof of Proposition 5.2

Eq (5.6) shows that the retailer’s optimal sales effort level e* is not related to the confidence level *η*.

According to Eq (5.5), take the first-order derivative of q* with respect to *η*:

$$\frac{\partial q^{*}}{\partial \eta }=\frac{p+\left(1-\beta \right)Y-w+\tau }{\left[p+\left(1-\beta \right)Y-s+\tau \right]\phi \left(q^{*}-z\left(e^{*}\right)\right)}>0$$

So q* increases with respect to *η*.

### A9: Proof of Proposition 5.3

According to Eq (5.6) and using the implicit function theorem, we denote

$$G_{2}\left(\tau ,e^{*}\right)=\left[p+\left(1-\beta \right)Y-w+\tau \right]z\prime \left(e^{*}\right)-\theta e^{*}$$

Taking the first derivative of e* with respect to *τ*, we obtain

$$\frac{\partial e^{*}}{\partial \tau }=-\frac{\partial G_{2}\left(\tau ,e^{*}\right)/\partial \tau }{\partial G_{2}\left(\tau ,e^{*}\right)/\partial e^{*}}=-\frac{z\prime \left(e^{*}\right)}{\left[p+\left(1-\beta \right)Y-w+\tau \right]z\prime \prime \left(e^{*}\right)-\theta }>0$$

Therefore e* increases with respect to the unit rebate/penalty *τ*.

According to Eq (5.5), taking the first derivative of q* with respect to *τ*, we obtain

$$\frac{\partial q^{*}}{\partial \tau }=\frac{\eta \left(w-s\right)}{\phi \left(q^{*}-z\left(e^{*}\right)\right){\left[p+\left(1-\beta \right)Y-s+\tau \right]}^{2}}+z\prime \left(e^{*}\right)\frac{\partial e^{*}}{\partial \tau }>0$$

Therefore q* also increases with respect to the unit rebate/penalty *τ*.

### A10: Proof of Theorem 5.2

As mentioned at the very beginning of Subsection 5.2, in order to maximize the supply chain profit, the SS-SRP contract which can achieve coordination should ensure that the retailer makes individual decisions aligning with the optimal centralized solutions of the integrated supply chain.

Comparing Eqs (3.3) and (5.6), and letting $e^{*}=e_{s}^{*}$, we have

p+(1−β)Y−w+τ=p+Y−c

Comparing Eqs (3.2) and (4.5), and letting $q^{*}=q_{s}^{*}$, we have

$$\frac{\eta \left[p+\left(1-\beta \right)Y-w+\tau \right]}{p+\left(1-\beta \right)Y-s+\tau }=\frac{p+Y-c}{p+Y-s}$$

Combining the above two equalities, we have

$$\begin{array}{ll} \{\begin{array}{l} p+\left(1-\beta \right)Y-w+\tau =p+Y-c\\ \frac{\eta \left[p+\left(1-\beta \right)Y-w+\tau \right]}{p+\left(1-\beta \right)Y-s+\tau }=\frac{p+Y-c}{p+Y-s} \end{array} & \Rightarrow \{\begin{array}{l} p+\left(1-\beta \right)Y-w+\tau =p+Y-c\\ p+\left(1-\beta \right)Y-s+\tau =\eta \left(p+Y-s\right) \end{array}\\ & \Rightarrow \{\begin{array}{l} w=c-\left(1-\eta \right)\left(p+Y-s\right)\\ \tau =\beta Y-\left(1-\eta \right)\left(p+Y-s\right) \end{array} \end{array}$$

### A11: Proof of Proposition 5.4

According to Eq (5.7), take the first-order derivative of *w* with respect to *η*:

$$\frac{\partial w}{\partial \eta }=p+Y-s>0$$

According to Eq (5.8), take the first-order derivative of *τ* with respect to *η*:

$$\frac{\partial \tau }{\partial \eta }=p+Y-s>0$$

So both *w* and *τ* increase with respect to *η*.

### A12: Proof of Proposition 5.5

The retailer’s optimal sales effort level under the SS-WP contract $e_{w}^{*}$ is given by Eq (4.6). A necessary condition for both players accepting the SS-WP contract is *w*_*w*_ + *β*_*w*_*Y* − *c* > 0, thus there is *p* + (1 − *β*_*w*_)*Y* − *w*_*w*_ < *p* + *Y* − *c*.

The retailer’s optimal sales effort level under the SS-SRP contract e* is given by Eq (5.6). According to the coordination parameters shown in Theorem 5.2, *p* + (1 –*β*)*Y*–*w* + *τ* = *p* + *Y* − *c*.

In order to compare $e_{w}^{*}$ and e*, denote *az*′(e*)–θe* = 0, where *a* > 0. Then we have

$$\frac{\partial e^{*}}{\partial a}=-\frac{z\prime \left(e^{*}\right)}{az\prime \prime \left(e^{*}\right)-\theta }>0$$

It means e* is increasing with respect to *a*. As *p* + (1 –*β*_*w*_)*Y*–*w*_*w*_ < *p* + *Y*–*c*, we obtain $e^{*}>e_{w}^{*}$.

According to Eq (4.5), the retailer’s optimal order quantity under the SS-WP contract $q_{w}^{*}$ can be expressed as follows.

$$q_{w}^{*}=\Phi^{-1}\left(\frac{\eta \left[p+\left(1-\beta_{w}\right)Y-w_{w}\right]}{p+\left(1-\beta_{w}\right)Y-s}\right)+z\left(e_{w}^{*}\right)=\Phi^{-1}\left(\eta \left[1-\frac{w_{w}-s}{p+\left(1-\beta_{w}\right)Y-s}\right]\right)+z\left(e_{w}^{*}\right)$$

According to Eq (5.5), the retailer’s optimal order quantity under the SS-SRP coordination contract q* can be expressed as follows.

$$q^{*}=\Phi^{-1}\left(\frac{\eta \left[p+\left(1-\beta \right)Y-w+\tau \right]}{p+\left(1-\beta \right)Y-s+\tau }\right)+z\left(e^{*}\right)=\Phi^{-1}\left(1-\frac{c-s}{p+Y-s}\right)+z\left(e^{*}\right)$$

When *w*_*w*_ + *β*_*w*_*Y* − *c* > 0, we have

$$\begin{array}{l} \frac{w_{w}-s}{p+\left(1-\beta_{w}\right)Y-s}-\frac{c-s}{p+Y-s}=\frac{\left(w_{w}-s\right)\left(p+Y-s\right)-\left(c-s\right)\left[p+\left(1-\beta_{w}\right)Y-s\right]}{\left[p+\left(1-\beta_{w}\right)Y-s\right]\left(p+Y-s\right)}\\ =\frac{\left(w_{w}-c\right)\left(p+Y-s\right)+\left(c-s\right)\beta_{w}Y}{\left[p+\left(1-\beta_{w}\right)Y-s\right]\left(p+Y-s\right)}>\frac{\left(w_{w}-c\right)\left(c-s\right)+\left(c-s\right)\beta_{w}Y}{\left[p+\left(1-\beta_{w}\right)Y-s\right]\left(p+Y-s\right)}=\frac{\left(w_{w}+\beta_{w}Y-c\right)\left(c-s\right)}{\left[p+\left(1-\beta_{w}\right)Y-s\right]\left(p+Y-s\right)}>0 \end{array}$$

Therefore,

$$\begin{array}{l} \frac{w_{w}-s}{p+\left(1-\beta_{w}\right)Y-s}>\frac{c-s}{p+Y-s}\Rightarrow 1-\frac{w_{w}-s}{p+\left(1-\beta_{w}\right)Y-s}<1-\frac{c-s}{p+Y-s}\\ \Rightarrow \Phi^{-1}\left(1-\frac{w_{w}-s}{p+\left(1-\beta_{w}\right)Y-s}\right)<\Phi^{-1}\left(1-\frac{c-s}{p+Y-s}\right)\Rightarrow \Phi^{-1}\left(\eta \left[1-\frac{w_{w}-s}{p+\left(1-\beta_{w}\right)Y-s}\right]\right)<\Phi^{-1}\left(1-\frac{c-s}{p+Y-s}\right) \end{array}$$

As $e^{*}>e_{w}^{*}$, there is $z\left(e^{*}\right)>z\left(e_{w}^{*}\right)$. We obtain

$$\Phi^{-1}\left(1-\frac{c-s}{p+Y-s}\right)+z\left(e^{*}\right)>\Phi^{-1}\left(\eta \left[1-\frac{w_{w}-s}{p+\left(1-\beta_{w}\right)Y-s}\right]\right)+z\left(e_{w}^{*}\right)$$

That is $q^{*}>q_{w}^{*}$.

### A13: Proof of Proposition 5.6

According to Eq (5.1), the retailer’s expected profit under the SS-SRP contract can be expressed as follows.

$$\begin{array}{ll} E\pi_{r}(q,e) & =\int_{0}^{q}\left[\left[p+\left(1-\beta \right)Y-s+\tau \right]x-\left(w-s\right)q-\left(\tau T+\theta e^{2}/2\right)\right]dF(x|e)\\ & +\int_{q}^{+\infty }\left[\left[p+\left(1-\beta \right)Y-w+\tau \right]q-\left(\tau T+\theta e^{2}/2\right)\right]dF(x|e)\\ & =\left[\left[p+\left(1-\beta \right)Y-w+\tau \right]q-\left(\tau T+\theta e^{2}/2\right)\right]F(q|e)-\left[p+\left(1-\beta \right)Y-s+\tau \right]\int_{0}^{q}F(x|e)dx\\ & +\left[\left[p+\left(1-\beta \right)Y-w+\tau \right]q-\left(\tau T+\theta e^{2}/2\right)\right]\left[1-F(q|e)\right]\\ & =\left[p+\left(1-\beta \right)Y-w+\tau \right]q-\left[p+\left(1-\beta \right)Y-s+\tau \right]\int_{0}^{q}F(x|e)dx-\tau T-\theta e^{2}/2 \end{array}$$

With the parameters presented in Theorem 5.2, the retailer’s optimal sales effort level and order quantity are equal to the centralized decisions of the integrated supply chain given by Theorem 3.1. So the retailer’s expected profit under the SS-SRP coordination contract can be expressed as follows.

$$\begin{array}{ll} E\pi_{r}(q_{s}^{*},e_{s}^{*}) & =\left[p+\left(1-\beta \right)Y-w+\tau \right]q_{s}^{*}-\left[p+\left(1-\beta \right)Y-s+\tau \right]\int_{0}^{q_{s}^{*}}F(x|e_{s}^{*})dx-\tau T-\theta e_{s}^{*2}/2\\ & =\left(p+Y-c\right)\left[\Phi^{-1}\left(\frac{p+Y-c}{p+Y-s}\right)+z\left(e_{s}^{*}\right)\right]-\eta \left(p+Y-s\right)\int_{0}^{\Phi^{-1}\left(\frac{p+Y-c}{p+Y-s}\right)+z\left(e_{s}^{*}\right)}\Phi \left(x-z\left(e_{s}^{*}\right)\right)dx-\tau T-\theta e_{s}^{*2}/2\\ & =\left(p+Y-c\right)\left[\Phi^{-1}\left(\frac{p+Y-c}{p+Y-s}\right)+z\left(e_{s}^{*}\right)\right]-\eta \left(p+Y-s\right)\int_{0}^{\Phi^{-1}\left(\frac{p+Y-c}{p+Y-s}\right)}\Phi \left(\xi \right)d\xi -\tau T-\theta e_{s}^{*2}/2 \end{array}$$ (A.3)

With the retailer’s joint decision (*q*,*e*), the manufacturer’s expected profit under the SS-SRP contract *Eπ*_*m*_(*q*,*e*) can be expressed as follows.

$$\begin{array}{ll} E\pi_{m}(q,e) & =\int_{0}^{q}\left[\left(w-c\right)q+\left(\beta Y-\tau \right)x+\tau T\right]dF(x|e)+\int_{q}^{+\infty }\left[\left(w+\beta Y-c-\tau \right)q+\tau T\right]dF(x|e)\\ & =\int_{0}^{q}\left[\left(w-c\right)q+\left(\beta Y-\tau \right)x+\tau T\right]dF(x|e)+\int_{q}^{+\infty }\left[\left(w+\beta Y-c-\tau \right)q+\tau T\right]dF(x|e)\\ & =\left(w+\beta Y-c-\tau \right)q-\left(\beta Y-\tau \right)\int_{0}^{q}F(x|e)dx+\tau T \end{array}$$

The manufacturer’s expected profit under the SS-SRP coordination contract can be expressed as follows.

$$\begin{array}{ll} E\pi_{m}(q_{s}^{*},e_{s}^{*}) & =\left(\beta Y-\tau -c+w\right)q_{s}^{*}-\left(\beta Y-\tau \right)\int_{0}^{q_{s}^{*}}F(x|e_{s}^{*})dx+\tau T\\ & =-\left(1-\eta \right)\left(p+Y-s\right)\int_{0}^{\Phi^{-1}\left(\frac{p+Y-c}{p+Y-s}\right)+z\left(e_{s}^{*}\right)}\Phi \left(x-z\left(e_{s}^{*}\right)\right)dx+\tau T\\ & =\tau T-\left(1-\eta \right)\left(p+Y-s\right)\int_{0}^{\Phi^{-1}\left(\frac{p+Y-c}{p+Y-s}\right)}\Phi \left(\xi \right)d\xi \end{array}$$ (A.4)

The total expected profit of the supply chain can be expressed as follows.

$$\begin{array}{l} E\pi_{r}(q_{s}^{*},e_{s}^{*})+E\pi_{m}(q_{s}^{*},e_{s}^{*})\\ =\left(p+Y-c\right)\left[\Phi^{-1}\left(\frac{p+Y-c}{p+Y-s}\right)+z\left(e_{s}{}^{*}\right)\right]-\left(p+Y-s\right)\int_{0}^{\Phi^{-1}\left(\frac{p+Y-c}{p+Y-s}\right)}\Phi \left(\xi \right)d\xi -\theta e_{s}^{*2}/2\\ =\left(p+Y-c\right)\left[\Phi^{-1}\left(\frac{p+Y-c}{p+Y-s}\right)+z\left(e_{s}^{*}\right)\right]-\left(p+Y-s\right)\left[\Phi^{-1}\left(\frac{p+Y-c}{p+Y-s}\right)\frac{p+Y-c}{p+Y-s}-\int_{0}^{\Phi^{-1}\left(\frac{p+Y-c}{p+Y-s}\right)}\xi d\Phi \left(\xi \right)\right]-\theta e_{s}^{*2}/2\\ =\left(p+Y-c\right)z\left(e_{s}^{*}\right)+\left(p+Y-s\right)\int_{0}^{\Phi^{-1}\left(\frac{p+Y-c}{p+Y-s}\right)}\xi d\Phi \left(\xi \right)-\theta e_{s}^{*2}/2 \end{array}$$ (A.5)

Comparing Eq (A.5) with Eq (3.4), obviously there is $E\pi_{s}(q_{s}^{*},e_{s}^{*})=E\pi_{r}(q_{s}^{*},e_{s}^{*})+E\pi_{m}(q_{s}^{*},e_{s}^{*})$. Thus with $q_{s}^{*}$ and $e_{s}^{*}$ determined, the expected profit of the NEV supply chain can be arbitrarily allocated between the retailer and the manufacturer by varying *T*.

### A14: Proof of Proposition 5.7

According to Eq (A.3), take the first-order derivative of $E\pi_{r}(q_{s}^{*},e_{s}^{*})$ with respect to *η*:

$$\frac{\partial E\pi_{r}(q_{s}^{*},e_{s}^{*})}{\partial \eta }=-\left(p+Y-s\right)\int_{0}^{\Phi^{-1}\left(\frac{p+Y-c}{p+Y-s}\right)}\Phi \left(\xi \right)d\xi -\left(p+Y-s\right)T=-\left(p+Y-s\right)\left[\int_{0}^{\Phi^{-1}\left(\frac{p+Y-c}{p+Y-s}\right)}\Phi \left(\xi \right)d\xi +T\right]<0$$

So $E\pi_{r}(q_{s}^{*},e_{s}^{*})$ decreases with respect to *η*.

According to Eq (A.4), take the first-order derivative of $E\pi_{m}(q_{s}^{*},e_{s}^{*})$ with respect to *η*:

$$\frac{\partial E\pi_{m}(q_{s}^{*},e_{s}^{*})}{\partial \eta }=\left(p+Y-s\right)T+\left(p+Y-s\right)\int_{0}^{\Phi^{-1}\left(\frac{p+Y-c}{p+Y-s}\right)}\Phi \left(\xi \right)d\xi =\left(p+Y-s\right)\left[T+\int_{0}^{\Phi^{-1}\left(\frac{p+Y-c}{p+Y-s}\right)}\Phi \left(\xi \right)d\xi \right]>0$$

So $E\pi_{m}(q_{s}^{*},e_{s}^{*})$ increases with respect to *η*.

### A15: Proof of Proposition 5.8

With and without the sales effort, the change of the retailer’s expected profit $\Delta E\pi_{r}\left(e_{s}^{*}\right)$ can be obtained by ordering $e_{s}^{*}=0$ in Eq (A.3), presented as follows.

$$\Delta E\pi_{r}\left(e_{s}^{*}\right)=\left(p+Y-c\right)z\left(e_{s}^{*}\right)-\theta e_{s}^{*2}/2$$ (A.6)

Similarly, the change of the manufacturer’s expected profit with and without the retailer’s sales effort can be obtained from Eq (A.4):

$$\Delta E\pi_{m}\left(e_{s}^{*}\right)=0$$ (A.7)

The change of the entire supply chain’s expected profit with and without the retailer’s sales effort $\Delta E\pi_{s}\left(e_{s}^{*}\right)$ is obtained by ordering $e_{s}^{*}=0$ in Eq (3.4):

$$\Delta E\pi_{s}\left(e_{s}^{*}\right)=\left(p+Y-c\right)z\left(e_{s}^{*}\right)-\theta e_{s}^{*2}/2$$ (A.8)

As $\Delta E\pi_{m}\left(e_{s}^{*}\right)=0$ and $\Delta E\pi_{r}\left(e_{s}^{*}\right)=\Delta E\pi_{s}\left(e_{s}^{*}\right)$, the retailer occupies all the profit increase generated by his sales effort and the manufacturer doesn’t benefit from the retailer’s sales effort.

## Supporting information

S1 Fig The SS-SRP contract faced by retailer. (TIFF)

S2 Fig Impact of *Y* on the order quantities (*η* = 0.9, *σ* = 200). (FIG)

S3 Fig Impact of *η* on the order quantities (*Y =* 40000, *σ* = 200). (FIG)

S4 Fig Impact of *σ* on the order quantities (*Y =* 40000, *η* = 0.9). (FIG)

S5 Fig Impact of *T* on the profit allocation (*Y =* 40000, *η* = 0.9, *σ* = 200). (FIG)

S6 Fig Impact of *Y* on the profit allocation (*T* = 500, *η* = 0.9, *σ* = 200). (FIG)

S7 Fig Impact of *η* on the profit allocation (*T* = 500, *Y =* 40000, *σ* = 200). (FIG)

S8 Fig Impact of *σ* on the profit allocation (*T* = 500, *Y =* 40000, *η* = 0.9). (FIG)

S1 File Numerical experiments code (matlab). (DOCX)
