# Supplementary material for: NEV supply chain coordination and sustainability considering sales effort and risk aversion under the CVaR criterion
Source: PLoS One. 2018 Jun 18;13(6):e0199005. doi: 10.1371/journal.pone.0199005 (PMC6005517; doi:10.1371/journal.pone.0199005)

**Numerical experiments code (Matlab)**

**for**

**NEV Supply Chain Coordination and Sustainability Considering Sales Effort and Risk Aversion under the CVaR Criterion**

**c=100000 p=80000 s=40000 $\beta=0.8$ $z\left(e\right)=e$ $\theta=100$ $\xi$~N(1000,σ)**

**Fig 2、Impact of $Y$ on the order quantities ($\eta=0.9$,** $\sigma=200$**)**

clear

p=80000;c=100000;s=40000;n=0.9;b=0.8;h=100; w=84000;

fori=20:60

x(i)=i*1000;

e1(i)=(p+(1-b)*x(i)-w)/h;

y1(i)=norminv(n*(p+(1-b)*x(i)-w)/(p+(1-b)*x(i)-s),1000,200)+e1(i);

e2(i)=(p+x(i)-c)/h;

y2(i)=norminv((p+x(i)-c)/(p+x(i)-s),1000,200)+e2(i);

end

plot(x,y1,'b',x,y2,'g')

axis([20000,60000,400,1400]);

xlabel('government subsidies Y')

ylabel('order quantities')


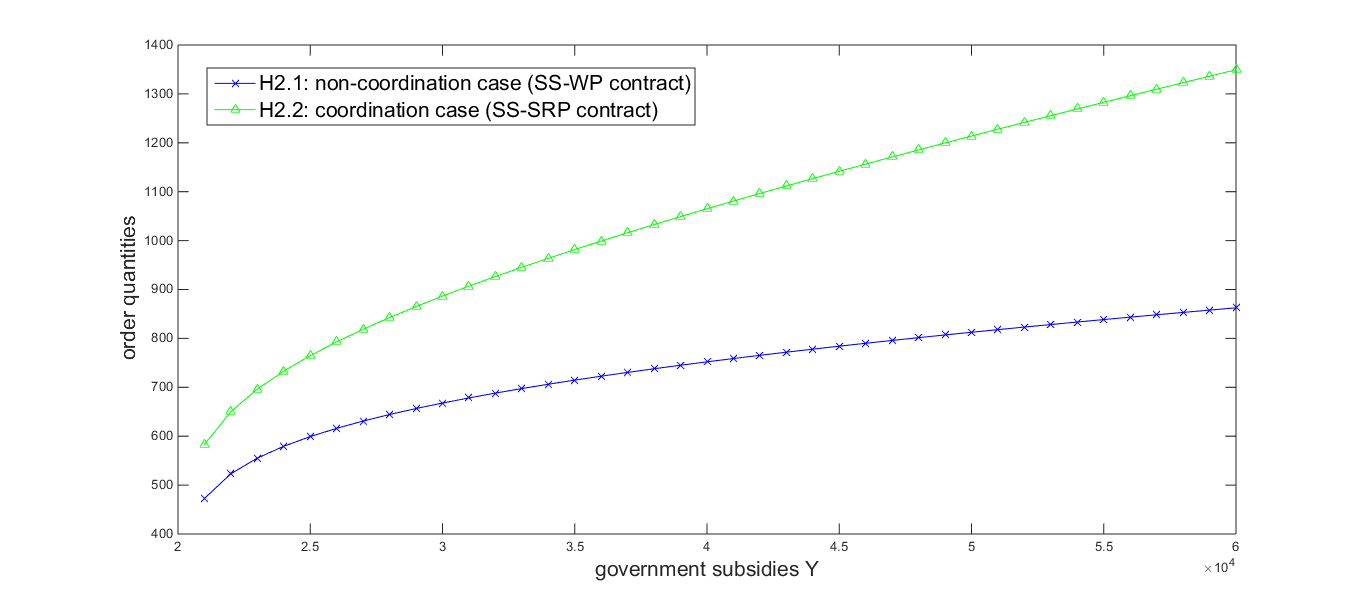


**Fig 3、Impact of $\eta$ on the order quantities (**$Y=40000$**,** $\sigma=200$**)**

clear

p=80000;Y=40000;c=100000;s=40000;b=0.8;h=100;w=84000;

fori=1:51

x(i)=(i-1)*0.02;

e1=(p+(1-b)*Y-w)/h;

y1(i)=norminv(x(i)*(p+(1-b)*Y-w)/(p+(1-b)*Y-s),1000,200)+e1;

e2=(p+Y-c)/h;

y2(i)=norminv((p+Y-c)/(p+Y-s),1000,200)+e2+0*x(i);

end

plot(x,y1,'b',x,y2,'g')

axis([0,1,400,1300]);

xlabel('retailer’s confidence level \eta')

ylabel('order quantities')


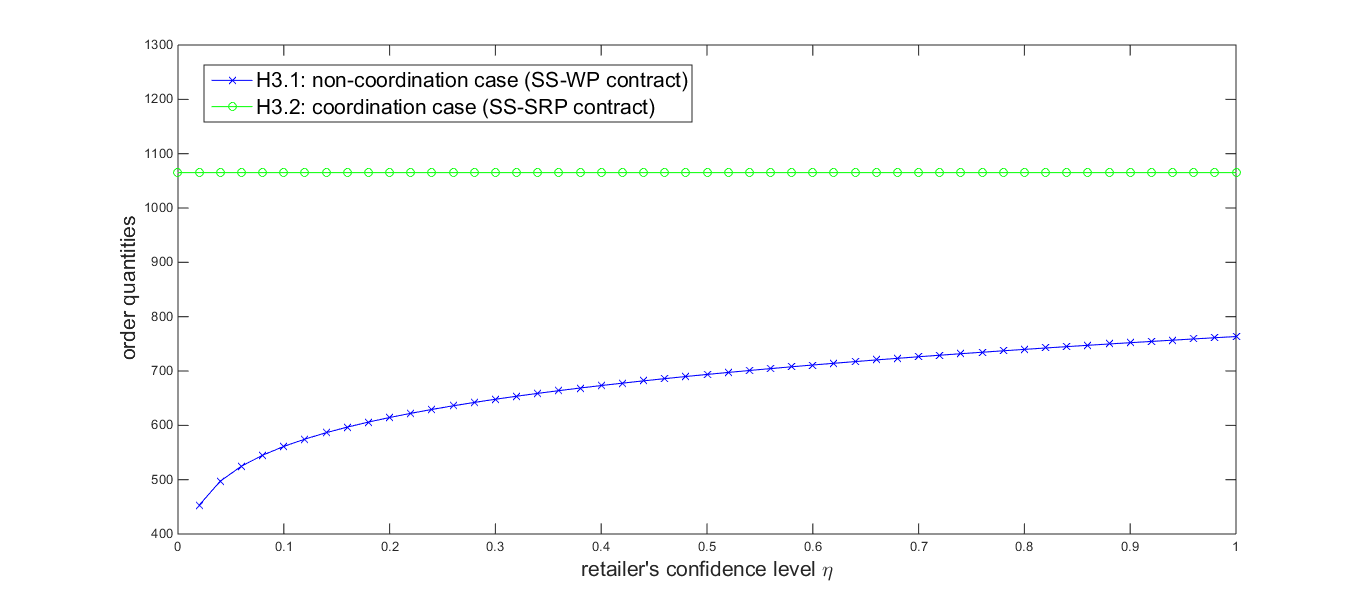


**Fig 4、Impact of $\sigma$ on the order quantities (**$Y=40000$**, $\eta=0.9$)**

clear

p=80000;Y=40000;c=100000;s=40000;n=0.9;b=0.8;h=100;w=84000;

fori=1:31

x(i)=(i-1)*20;

e1=(p+(1-b)*Y-w)/h;

y1(i)=norminv(n*(p+(1-b)*Y-w)/(p+(1-b)*Y-s),1000,x(i))+e1;

e2=(p+Y-c)/h;

y2(i)=norminv((p+Y-c)/(p+Y-s),1000,x(i))+e2;

end

plot(x,y1,'b',x,y2,'g')

axis([0,600, 0,1400]);

xlabel('demand standard deviation \sigma')

ylabel('order quantities')

**
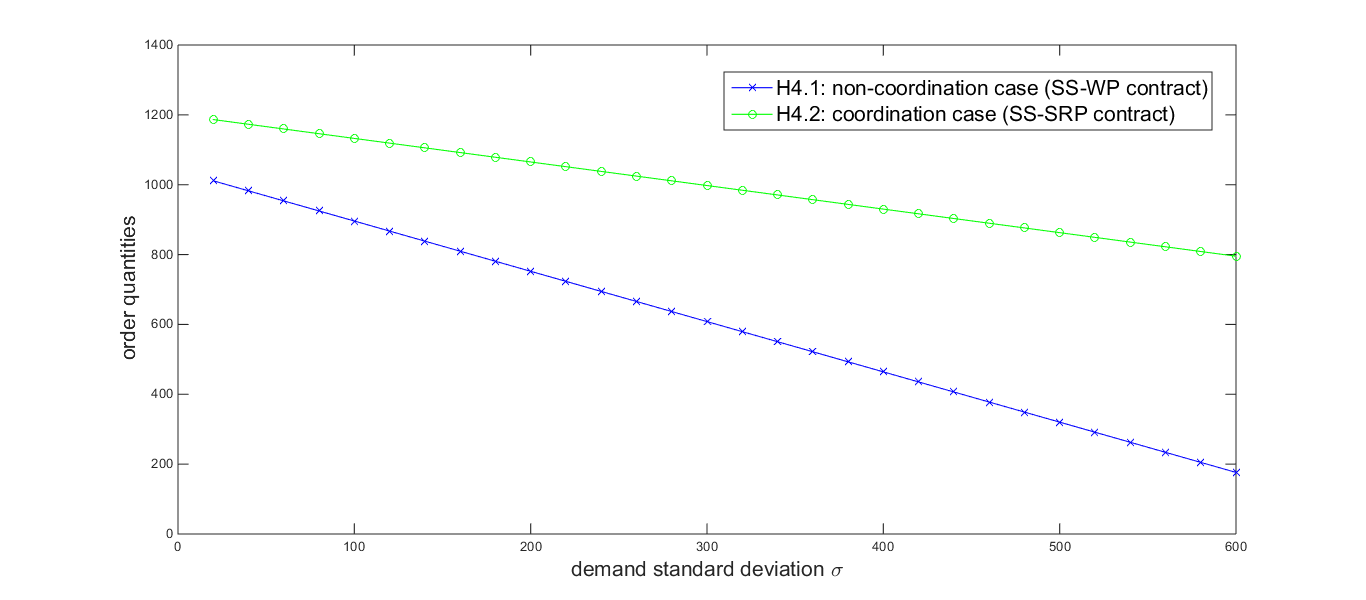
**

**Fig 5、Impact of $T$ on the profit allocation ($Y=40000$, $\eta=0.9$,** $\sigma=200$**)**

clear

p=80000;Y=40000;c=100000;s=40000;n=0.9;b=0.8;h=100;w=84000;

e1=(p+(1-b)*Y-w)/h;

e2=(p+Y-c)/h;

t=b*Y-(1-n)*(p+Y-s);

x=0:20:1000;

y1=(p+(1-b)*Y-w)*(norminv(n*(p+(1-b)*Y-w)/(p+(1-b)*Y-s),1000,200)+e1)-(p+(1-b)*Y-s)*(norminv(n*(p+(1-b)*Y-w)/(p+(1-b)*Y-s),1000,200)*n*(p+(1-b)*Y-w)/(p+(1-b)*Y-s)-1000*n*(p+(1-b)*Y-w)/(p+(1-b)*Y-s)+200*200*normpdf(norminv(n*(p+(1-b)*Y-w)/(p+(1-b)*Y-s),1000,200),1000,200))-h*e1*e1/2+0*x;

y2=(w+b*Y-c)*(norminv(n*(p+(1-b)*Y-w)/(p+(1-b)*Y-s),1000,200)+e1)-b*Y*(norminv(n*(p+(1-b)*Y-w)/(p+(1-b)*Y-s),1000,200)*n*(p+(1-b)*Y-w)/(p+(1-b)*Y-s)-1000*n*(p+(1-b)*Y-w)/(p+(1-b)*Y-s)+200*200*normpdf(norminv(n*(p+(1-b)*Y-w)/(p+(1-b)*Y-s),1000,200),1000,200))+0*x;

y3=y1+y2;

y4=(p+Y-c)*(norminv((p+Y-c)/(p+Y-s),1000,200)+e2)-n*(p+Y-s)*(norminv((p+Y-c)/(p+Y-s),1000,200)*(p+Y-c)/(p+Y-s)-1000*(p+Y-c)/(p+Y-s)+200*200*normpdf(norminv((p+Y-c)/(p+Y-s),1000,200),1000,200))-t*x-h*e2*e2/2;

y5=t*x-(1-n)*(p+Y-s)*(norminv((p+Y-c)/(p+Y-s),1000,200)*(p+Y-c)/(p+Y-s)-1000*(p+Y-c)/(p+Y-s)+200*200*normpdf(norminv((p+Y-c)/(p+Y-s),1000,200),1000,200));

y6=y4+y5;

plot(x,y1,'b',x,y2,'g',x,y3,'r',x,y4,'b',x,y5,'g',x,y6,'r')

axis([0,1000,-10000000,45000000]);

xlabel('sales target T')

ylabel('profit allocation')


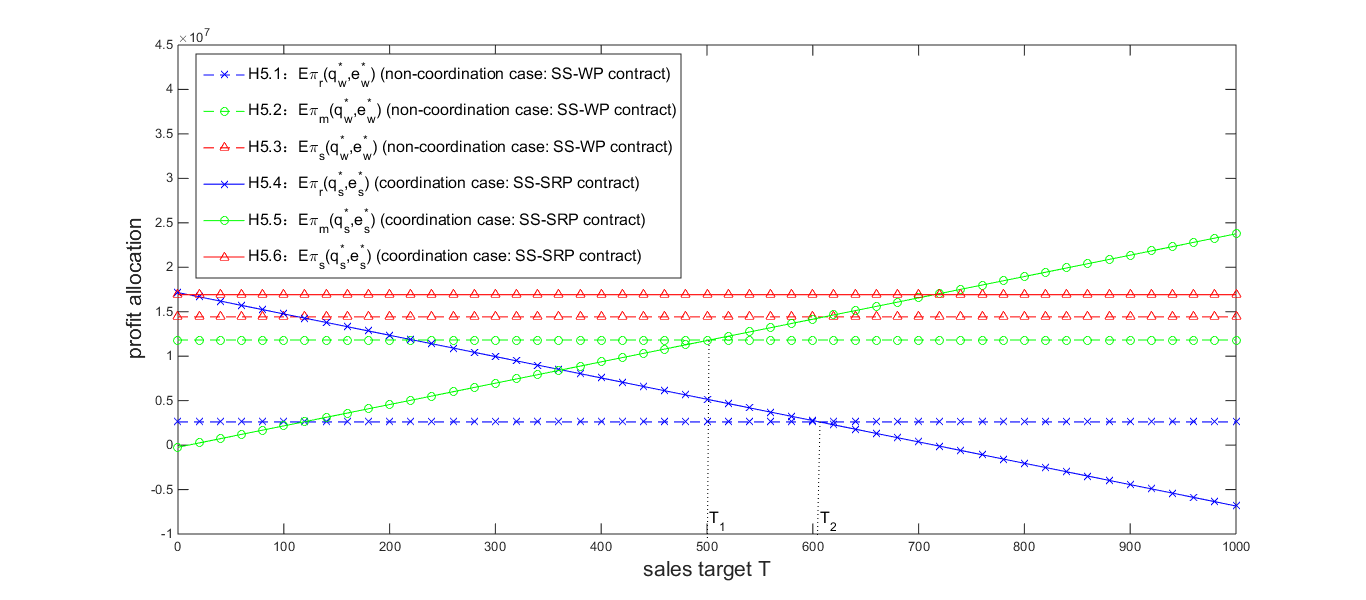


**Fig 6、Impact of $Y$ on the profit allocation ($T=500$, $\eta=0.9$,** $\sigma=200$**)**

clear

p=80000;c=100000;s=40000;n=0.9;b=0.8;h=100;T=500;w=84000;

fori=20:60

x(i)=i*1000;

e1(i)=(p+(1-b)*x(i)-w)/h;

y1(i)=(p+(1-b)*x(i)-w)*(norminv(n*(p+(1-b)*x(i)-w)/(p+(1-b)*x(i)-s),1000,200)+e1(i))-(p+(1-b)*x(i)-s)*(norminv(n*(p+(1-b)*x(i)-w)/(p+(1-b)*x(i)-s),1000,200)*n*(p+(1-b)*x(i)-w)/(p+(1-b)*x(i)-s)-1000*n*(p+(1-b)*x(i)-w)/(p+(1-b)*x(i)-s)+200*200*normpdf(norminv(n*(p+(1-b)*x(i)-w)/(p+(1-b)*x(i)-s),1000,200),1000,200))-h*e1(i)*e1(i)/2;

y2(i)=(w+b*x(i)-c)*(norminv(n*(p+(1-b)*x(i)-w)/(p+(1-b)*x(i)-s),1000,200)+e1(i))-b*x(i)*(norminv(n*(p+(1-b)*x(i)-w)/(p+(1-b)*x(i)-s),1000,200)*n*(p+(1-b)*x(i)-w)/(p+(1-b)*x(i)-s)-1000*n*(p+(1-b)*x(i)-w)/(p+(1-b)*x(i)-s)+200*200*normpdf(norminv(n*(p+(1-b)*x(i)-w)/(p+(1-b)*x(i)-s),1000,200),1000,200));

y3(i)=y1(i)+y2(i);

e2(i)=(p+x(i)-c)/h;

t(i)=b*x(i)-(1-n)*(p+x(i)-s);

y4(i)=(p+x(i)-c)*(norminv((p+x(i)-c)/(p+x(i)-s),1000,200)+e2(i))-n*(p+x(i)-s)*(norminv((p+x(i)-c)/(p+x(i)-s),1000,200)*(p+x(i)-c)/(p+x(i)-s)-1000*(p+x(i)-c)/(p+x(i)-s)+200*200*normpdf(norminv((p+x(i)-c)/(p+x(i)-s),1000,200),1000,200))-t(i)*T-h*e2(i)*e2(i)/2;

y5(i)=t(i)*T-(1-n)*(p+x(i)-s)*(norminv((p+x(i)-c)/(p+x(i)-s),1000,200)*(p+x(i)-c)/(p+x(i)-s)-1000*(p+x(i)-c)/(p+x(i)-s)+200*200*normpdf(norminv((p+x(i)-c)/(p+x(i)-s),1000,200),1000,200));

y6(i)=y4(i)+y5(i);

end

plot(x,y1,'b',x,y2,'g',x,y3,'r',x,y4,'b',x,y5,'g',x,y6,'r')

axis([20000,60000,-5000000,45000000]);

xlabel('government subsidies Y')

ylabel('profit allocation')


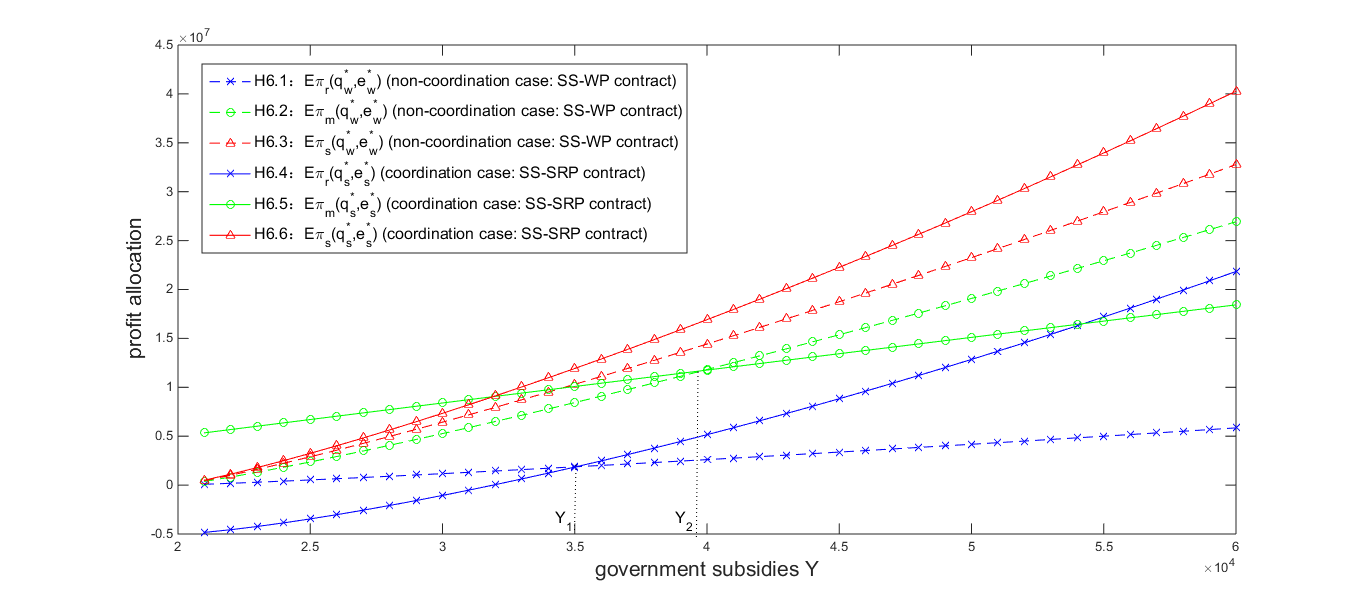


**Fig 7、Impact of $\eta$ on the profit allocation ($T=500$, $Y=40000$,** $\sigma=200$**)**

clear

p=80000;Y=40000;c=100000;s=40000;b=0.8;h=100;T=500;w=84000;

fori=1:51

x(i)=(i-1)*0.02;

e1=(p+(1-b)*Y-w)/h;

y1(i)=(p+(1-b)*Y-w)*(norminv(x(i)*(p+(1-b)*Y-w)/(p+(1-b)*Y-s),1000,200)+e1)-(p+(1-b)*Y-s)*(norminv(x(i)*(p+(1-b)*Y-w)/(p+(1-b)*Y-s),1000,200)*x(i)*(p+(1-b)*Y-w)/(p+(1-b)*Y-s)-1000*x(i)*(p+(1-b)*Y-w)/(p+(1-b)*Y-s)+200*200*normpdf(norminv(x(i)*(p+(1-b)*Y-w)/(p+(1-b)*Y-s),1000,200),1000,200))-h*e1*e1/2;

y2(i)=(w+b*Y-c)*(norminv(x(i)*(p+(1-b)*Y-w)/(p+(1-b)*Y-s),1000,200)+e1)-b*Y*(norminv(x(i)*(p+(1-b)*Y-w)/(p+(1-b)*Y-s),1000,200)*x(i)*(p+(1-b)*Y-w)/(p+(1-b)*Y-s)-1000*x(i)*(p+(1-b)*Y-w)/(p+(1-b)*Y-s)+200*200*normpdf(norminv(x(i)*(p+(1-b)*Y-w)/(p+(1-b)*Y-s),1000,200),1000,200));

y3(i)=y1(i)+y2(i);

e2=(p+Y-c)/h;

t(i)=b*Y-(1-x(i))*(p+Y-s);

y4(i)=(p+Y-c)*(norminv((p+Y-c)/(p+Y-s),1000,200)+e2)-x(i)*(p+Y-s)*(norminv((p+Y-c)/(p+Y-s),1000,200)*(p+Y-c)/(p+Y-s)-1000*(p+Y-c)/(p+Y-s)+200*200*normpdf(norminv((p+Y-c)/(p+Y-s),1000,200),1000,200))-t(i)*T-h*e2*e2/2;

y5(i)=t(i)*T-(1-x(i))*(p+Y-s)*(norminv((p+Y-c)/(p+Y-s),1000,200)*(p+Y-c)/(p+Y-s)-1000*(p+Y-c)/(p+Y-s)+200*200*normpdf(norminv((p+Y-c)/(p+Y-s),1000,200),1000,200));

y6(i)=y4(i)+y5(i);

end

plot(x,y1,'b',x,y2,'g',x,y3,'r',x,y4,'b',x,y5,'g',x,y6,'r')

axis([0,1,-30000000,60000000]);

xlabel('retailer’s confidence level \eta')

ylabel('profit allocation')


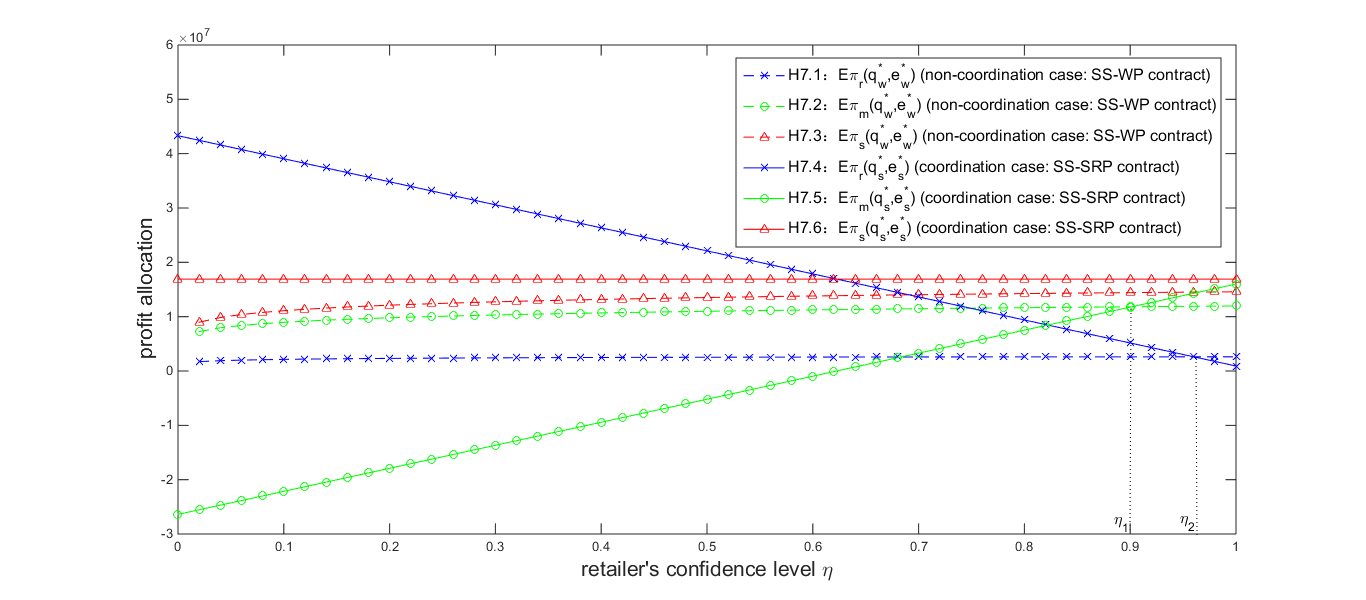


**Fig 8、Impact of $\sigma$ on the profit allocation ($T=500$, $Y=40000$, $\eta=0.9$)**

clear

p=80000;c=100000;s=40000;n=0.9;b=0.8;h=100;T=500;Y=40000;w=84000;

fori=1:51

x(i)=(i-1)*20;

e1=(p+(1-b)*Y-w)/h;

y1(i)=(p+(1-b)*Y-w)*(norminv(n*(p+(1-b)*Y-w)/(p+(1-b)*Y-s),1000,x(i))+e1)-(p+(1-b)*Y-s)*(norminv(n*(p+(1-b)*Y-w)/(p+(1-b)*Y-s),1000,x(i))*n*(p+(1-b)*Y-w)/(p+(1-b)*Y-s)-1000*n*(p+(1-b)*Y-w)/(p+(1-b)*Y-s)+x(i)*x(i)*normpdf(norminv(n*(p+(1-b)*Y-w)/(p+(1-b)*Y-s),1000,x(i)),1000,x(i)))-h*e1*e1/2;

y2(i)=(w+b*Y-c)*(norminv(n*(p+(1-b)*Y-w)/(p+(1-b)*Y-s),1000,x(i))+e1)-b*Y*(norminv(n*(p+(1-b)*Y-w)/(p+(1-b)*Y-s),1000,x(i))*n*(p+(1-b)*Y-w)/(p+(1-b)*Y-s)-1000*n*(p+(1-b)*Y-w)/(p+(1-b)*Y-s)+x(i)*x(i)*normpdf(norminv(n*(p+(1-b)*Y-w)/(p+(1-b)*Y-s),1000,x(i)),1000,x(i)));

y3(i)=y1(i)+y2(i);

e2=(p+Y-c)/h;

t=b*Y-(1-n)*(p+Y-s);

y4(i)=(p+Y-c)*(norminv((p+Y-c)/(p+Y-s),1000,x(i))+e2)-n*(p+Y-s)*(norminv((p+Y-c)/(p+Y-s),1000,x(i))*(p+Y-c)/(p+Y-s)-1000*(p+Y-c)/(p+Y-s)+x(i)*x(i)*normpdf(norminv((p+Y-c)/(p+Y-s),1000,x(i)),1000,x(i)))-t*T-h*e2*e2/2;

y5(i)=t*T-(1-n)*(p+Y-s)*(norminv((p+Y-c)/(p+Y-s),1000,x(i))*(p+Y-c)/(p+Y-s)-1000*(p+Y-c)/(p+Y-s)+x(i)*x(i)*normpdf(norminv((p+Y-c)/(p+Y-s),1000,x(i)),1000,x(i)));

y6(i)=y4(i)+y5(i);

end

plot(x,y1,'b',x,y2,'g',x,y3,'r',x,y4,'b',x,y5,'g',x,y6,'r')

axis([0,1000,-20000000,40000000]);

xlabel('demand standard deviation \sigma')

ylabel('profit allocation')


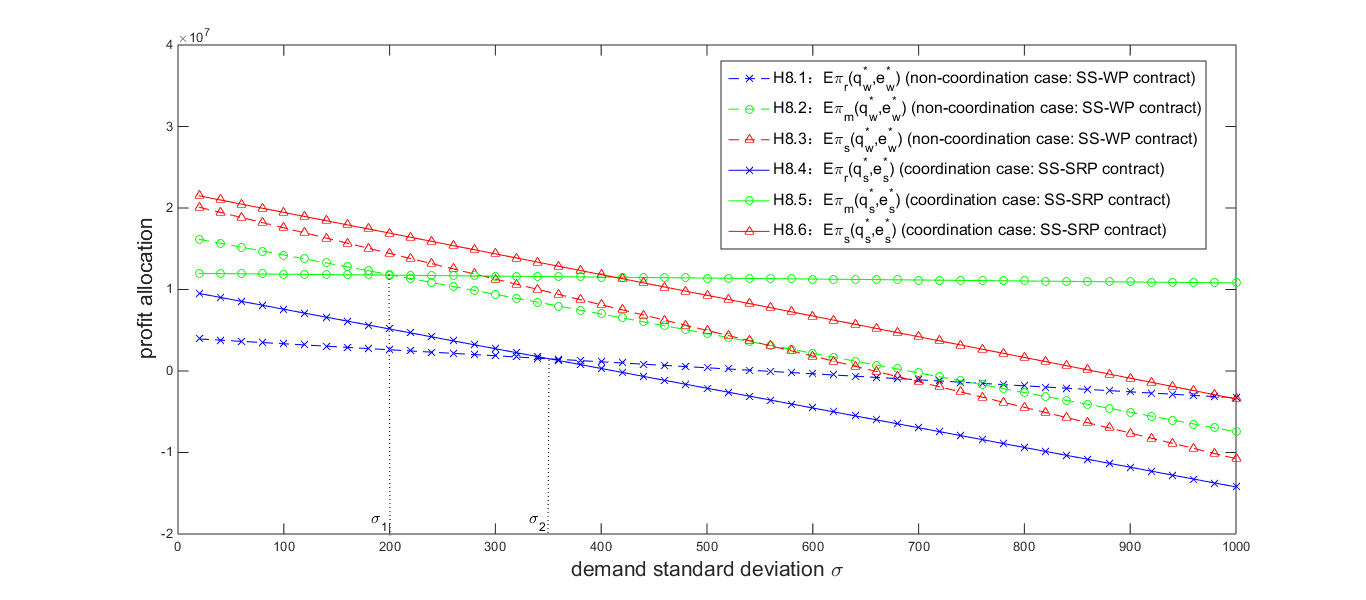

Supplement: S1 File — (DOCX) [file pone.0199005.s009.docx]
